# Changing Human Visual Field Organization from Early Visual to Extra-Occipital Cortex

**DOI:** 10.1371/journal.pone.0000452

**Published:** 2007-05-16

**Authors:** Anthony I. Jack, Gaurav H. Patel, Serguei V. Astafiev, Abraham Z. Snyder, Erbil Akbudak, Gordon L. Shulman, Maurizio Corbetta

**Affiliations:** 1 Neurology, Washington University in St. Louis Medical School, St. Louis, Missouri, United States of America; 2 Anatomy and Neurobiology, Washington University in St. Louis Medical School, St. Louis, Missouri, United States of America; 3 Radiology, Washington University in St. Louis Medical School, St. Louis, Missouri, United States of America; New York University, United States of America

## Abstract

**Background:**

The early visual areas have a clear topographic organization, such that adjacent parts of the cortical surface represent distinct yet adjacent parts of the contralateral visual field. We examined whether cortical regions outside occipital cortex show a similar organization.

**Methodology/Principal Findings:**

The BOLD responses to discrete visual field locations that varied in both polar angle and eccentricity were measured using two different tasks. As described previously, numerous occipital regions are both selective for the contralateral visual field and show topographic organization within that field. Extra-occipital regions are also selective for the contralateral visual field, but possess little (or no) topographic organization. A regional analysis demonstrates that this weak topography is not due to increased receptive field size in extra-occipital areas.

**Conclusions/Significance:**

A number of extra-occipital areas are identified that are sensitive to visual field location. Neurons in these areas corresponding to different locations in the contralateral visual field do not demonstrate any regular or robust topographic organization, but appear instead to be intermixed on the cortical surface. This suggests a shift from processing that is predominately *local* in visual space, in occipital areas, to *global*, in extra-occipital areas. Global processing fits with a role for these extra-occipital areas in selecting a spatial locus for attention and/or eye-movements.

## Introduction

### Background

Both single-unit studies in macaques and BOLD imaging studies in humans indicate that early visual areas show a precise topographic organization, such that a large portion of occipital cortex consists of a series of smooth and continuous representations of the contralateral visual field [1]–[3]. Outside occipital cortex, both single-unit studies in macaques [4]–[8] and BOLD imaging studies in humans [9]–[19] provide evidence for areas in parietal and frontal cortices that prefer contralateral to ipsilateral visual locations. However, these studies provide different perspectives on the visual field organization of these higher areas.

Single unit studies in macaques have clearly demonstrated changes in visual field organization moving from early visual to higher visual areas. Felleman and Van Essen [20] distinguish four categories of topographic organization in the monkey, varying from extremely precise and regular (V1), through intermediate (V2/V3), course and irregular (e.g. V3A, V4), and finally little or no discernible topography. The exact categorization of topographic organization of areas in macaque extra-occipital cortex with visual receptive fields remains to be definitively determined; however recent studies suggest these regions fall in the last two categories. Thus, while most neurons in lateral intraparietal, arcuate, and principal sulci (LIP, FEF and area 46) respond more strongly to stimuli presented in the contralateral visual field, i.e. show a contralateral preference [but see 21], neurons representing any given polar angle within the contralateral field are relatively evenly distributed across the cortical surface. Therefore, at best very coarse polar angle topography exists in these areas, with a tendency for some grouping of neurons that represent similar parts of the visual field, and/or a modest skew in the distribution of receptive fields across the cortical surface [4]–[8].

In humans, studies of visual field organization have tended to emphasize the presence of topographic organization in early visual [1]–[3], higher occipital [22]–[26] and extra-occipital areas [9]–[12]. There has been less focus on differences in visual field organization between areas (but see [27]–[29]). The reason is that studies that have compared more than two visual locations in humans have tended to rely on a model-based approach called ‘phase-encoding’. In phase-encoding studies, the BOLD response at each voxel is measured as the location of a stimulus is cyclically varied at a fixed frequency. The phase of the response then reflects the stimulus position that evokes the strongest response. Two limitations of phase-encoding, as compared to single unit studies, are: (i) phase-encoding only measures the part of the BOLD response that varies with visual location. In contrast, single units both measure signals that vary with visual location and signals that are independent of visual location. (ii) phase-encoding studies do not distinguish different profiles of responses across visual locations. For instance, single unit studies indicate quite different profiles of response for early visual and extra-occipital areas in the macaque. Summing unit responses over a small patch of cortex in macaque V1 would produce a strong response to stimuli at one visual location in the contralateral field and greatly diminished responses to all other visual locations. In contrast, summing unit responses over a small patch of cortex in macaque principal sulcus (area 46) would produce nearly equal responses to all locations in the contralateral visual field, and diminished responses for locations in the ipsilateral visual field. Phase encoding measurements cannot easily distinguish between these two profiles of response.

### Goals and Significance

**Figure 1:**
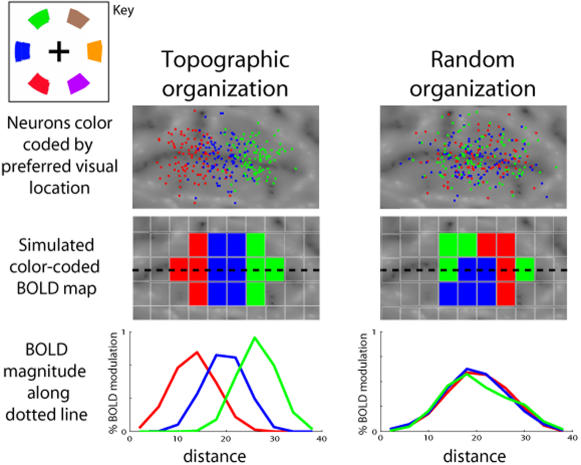
Different methods for assessing visual field organization. The top two panels show two simulated distributions of neurons overlaid on a portion of the right hemisphere's cortical surface. Note that all the neurons prefer locations in the contralateral (left) visual field (see figure key in top left corner). Electrophysiological studies consistently show that most neurons prefer contralateral locations. In the top left panel, the neurons are topographically organized, such that neurons preferring nearby visual locations tend to lie close to each other on the cortical surface. In the top right panel, there is no topographic organization, such that neurons preferring different parts of contralateral visual field are randomly intermixed on the cortical surface. The middle panels show simulated BOLD maps in which voxels are colored to indicate the preferred visual location. The topographic organization of the neurons on the left is accurately reflected in the surface map. However, the map on the right also produces an illusory impression of topography. The problem is that the maps show the best fitting location in a ‘winner take all’ fashion, even when the differences between locations are insignificant. A more in-depth examination requires looking at how the magnitude of BOLD response varies as we move across the cortical surface. The dotted black line illustrates a trajectory across the cortical surface. The graphs in the bottom panel plot BOLD magnitudes along that trajectory. The topographic and non-topographic cases can now be clearly distinguished. The investigations we report here employ measures that take account of the relative magnitude of BOLD response to distinct visual locations.

Figure 1 illustrates the sensitivity of different methods for detecting topographic organization. The goal of the current study is to use a methodology suited to revealing differences in visual field organization between areas. Images generated by phase encoding have created the impression of clear topographic organization in human extra-occipital cortex, akin to that seen in occipital visual areas. We wanted to assess whether this impression is correct. To do this, we obtained independent measurements of the BOLD response associated with discrete locations in the contralateral and ipsilateral visual fields, relative to a no-stimulus control. This technique allows us to measure (i) the magnitude of responses that depend on visual location vs. the magnitude of responses that do not; and (ii) measure the profile of response to different visual locations.

The ability to characterize visual field organization in this way is significant because it sheds light on the distribution of neuronal inputs to these areas [30]. This, in turn, has implications for the function of these areas [31]. It is also significant for methodological reasons. The ability to identify visual areas on the basis of their visual field organization has proven a key tool for neuroscientific research on the human visual system. The most efficient method for identifying specific visual areas will clearly depend on the nature of their visual field organization. A further motivation for this study is to help resolve an apparent discrepancy in current characterizations of visual field organization in human parietal lobe [9]–[11]. To address this, we initially focus our analysis by examining changes in visual field organization from early visual to parietal cortex. We then widen our net to examine other areas. In total we were able to identify seven extra-occipital regions sensitive to visual field location, all but one in cortical locations similar to where previous studies have found either topographic maps or a contralateral preference [10], [12]–[19].

### Experimental Design and Tasks

**Figure 2:**
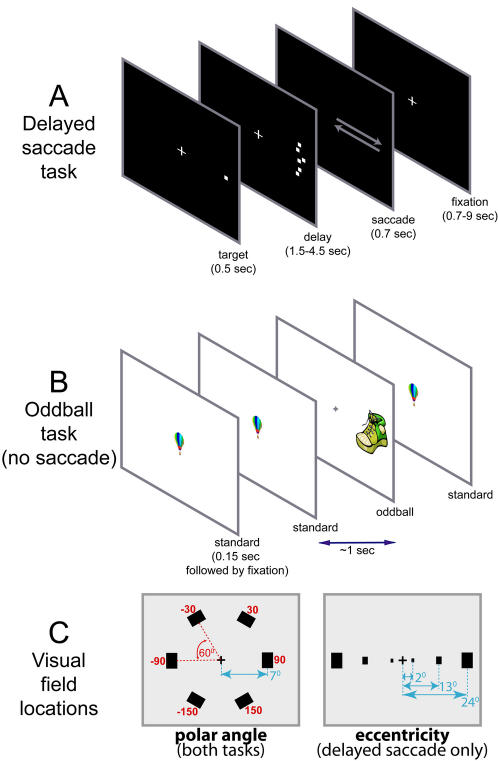
The two behavioral tasks used in the study. (A) In the delayed saccade task subjects maintained fixation while a target dot was briefly presented. This was followed by a variable delay period during which flickering dots appeared in the same sector of the visual field as the target. When the screen went black, subjects made a rapid saccade to the remembered target location, then back to the center. Trials occurred in blocks of three, with small variations in target location, within the same sector, between trials (see methods). (B) The oddball task required subjects to categorize each stimulus, presented approx. once per second, as either ‘standard’ or ‘oddball’ using a manual response. On most trials the same ‘standard’ object was presented at the center, while on 12% of trials a novel object was presented in one of 7 locations (fovea or 6 peripheral locations as in the polar angle version of the delayed saccade task). Subjects maintained fixation throughout. (C) In the oddball task and the polar angle version of the delayed saccade task, the six sector centers were evenly distributed around a circle with a radius of approx 7 degrees visual angle. In the eccentricity version of the delayed saccade task, the sector centers lay on the horizontal meridian, at 2, 13 or 24 degrees eccentricity.

We measured whole-brain BOLD response during performance of two different tasks. The primary task was a delayed saccade task, shown in Figure 2A. This task was similar to that used in recent phase-encoding studies of parietal cortex (with just one substantial difference, see below), in which subjects perform memory-guided saccades to different visual field locations [9], [10]. The second task was a visual oddball task, shown in Figure 2B. A stream of standard targets presented at the fovea was interrupted by unexpected and low frequency ‘oddball’ stimuli presented at different peripheral locations. This second task was originally chosen for its ability to activate a network of areas which are preferentially engaged when unexpected events occur and/or subjects are required to reorient attention [for a review see 32]. Hence, we expected this task to reveal visual field organization in additional areas, however the results turned out to be highly consistent across the two tasks. As a result, in our analyses the oddball task serves the role of an independent data set that is used to replicate findings from the delayed saccade task, while conveniently controlling for a variety of task-specific factors.

Since an overall goal of the experiment was to compare visual field organization in occipital and extra-occipital areas, we wanted to measure location specific responses in all areas using the same task. Previous studies have suggested a gradient of sensitivity, such that occipital regions are more sensitive to visual stimulation and extra-occipital regions are more sensitive to top-down factors, while all regions appear to show at least some sensitivity to both types of process [10], [11], [33], [34]. Thus, in the present tasks, visual stimulation and top-down processes were varied in tandem, allowing us to map both occipital and extra-occipital regions with maximal sensitivity using the same task. The trade-off is that no claims are made regarding the specific signal (e.g. sensory or attentional) that was mapped. The inclusion of visual stimulation, specific to the region of visual space as the saccade target, represented the only substantial difference between the delayed saccade task used here, and the tasks used in previous investigations [9], [10], [33].

A total of four subjects were tested. Their participation in different tasks is detailed in methods (Table 1). Since this study focuses on fine-grained functional anatomy, the data was not intentionally spatially smoothed at any point. Unavoidable smoothing due to co-registration of images and atlas registration was minimized by re-sampling the data only once (see methods).

**Table 1:** Number of scanning sessions (separate days) in which subjects participated in each task

Subject	Delayed saccade	Oddball task	Delayed saccade	Passive retinotopy
	*polar angle*	*polar angle*	*eccentricity*	
**A**	3	3	3	1
**B**	3	3	-	1
**C**	3	-	3	1
**D**	3	3	-	-

## Results

### Topographic maps of early visual areas

**Figure 3:**
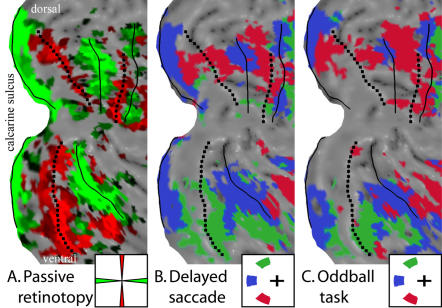
Correspondence between passive retinotopy and active mapping tasks for polar angle topography. A flattened representation of occipital cortex of subject A is shown. (A) shows data from a passive viewing task in which contrast reversing checkerboards were displayed in alternating blocks along the horizontal and vertical meridians. Lines corresponding to the most robust representations of the horizontal and vertical meridians were drawn on the basis of this data set, and are reproduced in the other panels for comparison. (B) shows data from the polar angle version of the delayed saccade paradigm. Voxels showing a preference for the contralateral field were colored according to which of the three contra-lateral locations produced the greatest response. Note the close correspondence for the horizontal meridian, and the slight gap in activated representation on the vertical meridian, due to the stimuli lying 30 degrees from the vertical in this paradigm. (C) shows data from the oddball task, derived in the same way.

We first show that our mapping technique yields the expected topography in early visual cortex (Figure 2). This provides preliminary support for the reliability of our methods. Our methods were designed to reveal visual field organization in both early and higher visual areas, by combining bottom-up and top-down influences. Since the topographic organization of early visual areas using passive viewing techniques has already been well documented, we sought to verify that we could reproduce the typical findings (Figure 3). In order to explicitly compare our active tasks with a traditional passive viewing of checkerboard wedges, we conducted meridian mapping in three of our subjects (see methods). The results are illustrated in Figure 3A. The solid and dotted black lines were drawn to mark the horizontal and vertical meridians on the basis of the passive data, and then overlaid on the data from the two active tasks (Figure 3B&C) to allow a close comparison. The horizontal meridian (solid line) from the meridian mapping task was centered within the middle-location stimuli from the active tasks, coded in dark blue. The vertical meridian (dotted line) from the meridian mapping task was slightly offset from the upper (green) and lower field (red) stimuli from the active tasks. This is exactly as would be predicted, since the upper and lower locations in the active tasks subtended 30 deg from the vertical meridian. The high degree of consistency between the tasks provides a validation of the underlying method and confirms that subjects maintained fixation during the active tasks.

### Progressive shift in organization from V1 to parietal cortex

**Figure 4:**
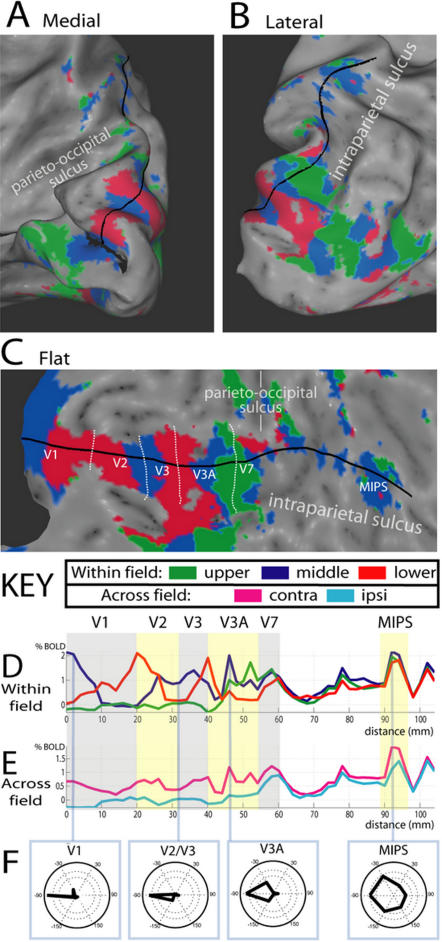
Visual field organization of dorsal visual areas and medial intra-parietal sulcus, from the polar angle version of the delayed saccade task. A and B: Medial and lateral views of an inflated representation of occipital cortex. C: flat representation of dorsal occipital and parietal cortex. Maps show preferred visual field location thresholded by contra-lateral preference, as in Figure 3 B&C. The cortical trajectory (shown in black) was drawn from the horizontal meridian of V1, in the calcarine sulcus, through V2, V3, V3A, V7 and then through those parts of medial intraparietal sulcus showing the greatest sensitivity to visual field location. D: Magnitude of BOLD activity associated with the three contra-lateral visual field positions along the trajectory (colors match maps in panels A–C). E: Mean magnitude associated with contra-lateral (pink) and ipsi-lateral (blue) visual field positions. F: Compass plots illustrating the BOLD magnitude associated with each of the six polar angles. Each plot comes from a cortical area demonstrating a preference for the contra-lateral horizontal meridian (blue regions in panels A–C). Data shown comes from the right hemisphere of subject A. Supplementary figures S1 and S2 show panels C–E for all eight hemispheres investigated.

We next aim to illustrate how visual field organization changes from early visual areas through to extra-occipital areas. The extra-occipital area we initially focus on is the medial bank of intra-parietal sulcus (IPS), since this has been a focus of prior investigations. Sereno et al [10] found evidence of an isolated region containing a map of the contralateral visual hemisphere some distance dorsal/anterior to V7, the most dorsal part of the retinotopic belt. This finding is supported by other studies that have found evidence of an isolated region of contralateral preference in a similar cortical location [e.g. 18], [19]. This region is sometimes referred to as the putative human homologue of monkey lateral intraparietal sulcus, or hLIP. In contrast, Silver et al [11] and Schluppeck et al [9] reported evidence for two topographic maps, IPS1 and IPS2, that tile the region extending anteriorly from V7 along the medial bank of IPS. Atlas coordinates appear to place hLIP anterior to both IPS1 and IPS2. One possibility, which might help account for these discrepancies, is that the functional organization of parietal regions sensitive to visual location may primarily reflect larger responses for contralateral than ipsilateral stimuli, i.e. contralateral organization, rather than differential responses within the contralateral field, i.e. topographic organization. If this were the case, the apparent topographic organization revealed by phase encoding maps would likely prove unreliable and misleading, causing different groups to reach divergent conclusions when drawing the borders between areas on the basis of those maps. To assess this possibility, we measured responses along a cortical trajectory from occipital cortex to IPS. Data from one hemisphere is illustrated in Figure 4. Data from all hemispheres can be found in figures S1 and S2.

### Path of the cortical trajectory

The single continuous ‘cortical trajectory’ is illustrated in Figures 4A–C. This trajectory was drawn by hand to optimally capture visual field organization in lower and higher occipital areas, and to focus on extra-occipital parietal regions investigated in earlier studies. The color-coded map in Figures 4A–C shows significantly greater activity for contralateral than ipsilateral locations, and is color-coded according to which of the three locations within the contralateral field produced the greatest BOLD response. Green corresponds to the upper visual field location, blue to the horizontal meridian location, and red to the lower visual field location (as in Figure 3B&C). The cortical trajectory started at the horizontal meridian representation of V1 in the fundus of the calcarine sulcus (Figures 4A and 4C), continued through dorsal visual areas V2, V3, V3A & V7, and the medial bank of IPS (Figure 4B). In visual areas, the trajectory was drawn to best capture the polar-angle topography that defines the borders between regions. It was then carefully extended into medial IPS to cross-sect those parts of cortex that showed the greatest evidence of spatial selectivity. Thus, this trajectory moved through the area of cortex between V7 and IPS previously reported to contain topographic maps IPS1 and IPS2 [9], [11]. Figure 4C shows this cortical trajectory on a flattened representation of cortex. Figures 4D&E shows BOLD responses to discrete visual field locations in the polar-angle version of the delayed saccade task, as a function of distance along the cortical surface. These are explained in further detail in the following sections.

### Responses to distinct contralateral locations

Evidence for progressive changes in topography is shown in Figure 4D, which plots the magnitude of the BOLD response to the three contralateral visual field locations at each point along the cortical trajectory. In early visual areas (V1, V2, V3) there was very little overlap in the activity due to different locations in the contralateral field, reflecting the fine topography in these areas. For instance, throughout dorsal V1–V3 there was very little activation for the upper field quadrant (green line); starting from the fundus of the calcarine sulcus (position 0 on the x-axis), high activity was measured for the horizontal meridian location (blue line) but this activity decreased as the stimulus moved toward the lower quadrant and the vertical meridian representation (red); correspondingly, the response for the lower quadrant (red line) increased to the maximal value (position 20 on x-axis). Similar reversals were seen in V2 and V3, which also contain quarter-field representations. As the cortical trajectory proceeded through V3A and V7, the responses to each location overlapped more, reflecting the coarser topography in those regions [20]. Finally, in parietal cortex (the swath of cortex between V7 and medial IPS, MIPS included) the BOLD responses to discrete contralateral locations were very similar, reflecting very coarse or absent topography. Even when the response functions separated; the spatial profile of activity remained very similar, indicating that the magnitudes associated with distinct contralateral field positions were highly correlated across the cortical surface.

A quantitative and statistical treatment of the visual impressions described here was derived by calculating correlation coefficients. This analysis fully supports the findings as described here and can be found in the supplementary text S1.

### Responses to contralateral and ipsilateral locations

Figure 4E plots the mean magnitudes of the response averaged over the three contralateral locations (pink line) and the response averaged over the three ipsilateral locations (light blue line). The ipsilateral response was negative or zero in V1–V3, but began to rise in V3A and V7 and was maintained throughout parietal cortex, reflecting an increase in the component of the BOLD signal that was independent of visual location. In parietal cortex immediately anterior/dorsal to V7, the ipsilateral response reached the level of the contralateral response, indicating a complete insensitivity to visual location; however a clear separation between contralateral and ipsilateral responses was observed for a small region in medial intraparietal sulcus. The location of this region in atlas space (Table 2) and relative to anatomical landmarks (supplementary text S2) suggests it was the same region identified by Sereno et al (2001), often referred to as hLIP. We refer to it as MIPS to avoid assumptions about human-monkey homology.

**Table 2:** Summary of extra-occipital cortical areas preferring the contra-lateral visual field.

Area	Hemi-sphere	Mean volume (mm^3^)	Talaraich coordinates mean(s.d.)			Mean peak contra-lateral z score		Number passing whole brain correction for delay saccade	Replication in oddball task	
			x	y	z	**Delay sacc.**	**Oddball**		**t**	**p**
**MIPS^v^**	L	347	**−30**(5)	**−58**(4)	**47**(4)	15.2	7.0	4/*4*	4.8	0.002
	R	261	**27**(1)	**−59**(6)	**52**(3)	15.7	5.7	4/*4*		
**PCu^v^**	L	171	**−7**(2)	**−60**(4)	**50**(2)	11.1	7.2	4/*4*	3.1	0.013
	R	117	**5**(3)	**−62**(11)	**52**(4)	9.8	3.8	4/*4*		
**ST^v^**	L	110	**−48**(3)	**−51**(9)	**6**(4)	6.6	3.6	2/*4*	3.8	0.006
	R	65	**56**(6)	**−39**(8)	**16**(5)	6.6	3.2	3/*4*		
**IFEF^v^**	L	234	**−34**(5)	**−11**(4)	**50**(5)	8.8	3.5	3/*4*	2.5	0.027
	R	214	**32**(4)	**−11**(3)	**52**(3)	9.9	3.9	4/*4*		
**SFEF^v^**	L	122	**−23**(3)	**−11**(6)	**55**(6)	6.2	3.4	3/*4*	4.0	0.005
	R	104	**21**(1)	**−11**(8)	**60**(6)	7.3	2.7	3/*4*		
**MPCe^v^**	L	203	**−46**(3)	**−5**(4)	**41**(4)	10.5	4.4	4/*4*	3.8	0.006
	R	97	**48**(7)	**−6**(5)	**38**(7)	7.8	3.0	2/*4*		
**IFS^v^**	L	180	**−40**(5)	**16**(6)	**28**(5)	7.7	3.0	3/*4*	3.2	0.011
	R	131	**39**(4)	**12**(4)	**29**(5)	8.4	3.8	3/*4*		

### Compass plots of spatial tuning

Figure 4F provides an overall plot of spatial tuning for polar angle in 4 areas. Within each area, the BOLD response is plotted across the six visual locations for a region that demonstrated a preference for the middle contralateral sector (colored blue in Figure 4A–C). Although these four regions showed the same overall location preference, the compass plots illustrated a progressive decrease in spatial tuning, with tight location tuning in V1, intermediate tuning in V3A and much coarser tuning in MIPS.

### Summary of findings from cortical trajectory analyses

The findings illustrated in Figure 4 were highly consistent across subjects (see figures S1 and S2). These analyses indicate a progressive change in visual field organization from early visual occipital areas to higher-order occipital areas to parietal cortex. Early visual areas responded highly selectively to stimuli presented at a specific contralateral location, i.e. good topography, with weak or negative responses to stimuli presented at ipsilateral locations. By IPS, however, topography was much weaker and ipsilateral responses were much stronger, indicating that a larger component of the BOLD signal was independent of visual location. Nonetheless, a region in IPS (MIPS) demonstrated a clear contralateral organization.

### Statistical reliability of MIPS across subjects and tasks

While previous studies of visual field organization in intraparietal cortex have reported partial and/or qualitative replications, they did not involve any formal test that the reported regions could be reliably identified. We regard this as an important step. We established the reliability of MIPS across hemispheres as follows. Statistical maps were computed for the contrast contralateral minus ipsilateral using data from the polar-angle version of the delayed saccade task and thresholded according to a conservative multiple-comparisons correction for the whole brain. We found a candidate region in the proximity of MIPS in every hemisphere (see mean coordinates in Table 2, and supplementary text S2 for a description of the anatomical landmarks). The average peak z-score in the delayed saccade task obtained by averaging the individual z-scores for the contra-ipsi contrast across hemispheres was 15.2 (Table 2).

We then tested the reliability of the MIPS regions across tasks by using data from the oddball task to test the reliability of the regions identified from the delayed saccade data. We computed for each individual the peak z-score for the contrast contralateral minus ipsilateral in the oddball task, i.e. oddball targets appearing in the contralateral vs. ipsilateral visual field, across all the voxels of the region, and then averaged this peak z-score over subjects and hemispheres. The mean replication z-score in the oddball task was 7.0 (Table 2). In addition, we performed a random effects analysis to establish that these apriori defined MIPS areas were reliable across the population of hemispheres as a whole, and were not driven by one or two robust examples. We computed response magnitudes for each region for the horizontal contralateral and ipsilateral stimulus positions in the oddball task. Six pairs of observations, one pair per hemisphere, were entered into a paired-t test. In this random-effect analysis, MIPS showed significantly stronger contralateral responses in the oddball task (t = 4.8 p = .002; see Table 2). These results demonstrate the statistical significance over hemispheres of a region in MIPS with a preference for the contralateral visual field.

### Additional extra-occipital areas sensitive to visual location

We used the replication approach shown above for MIPS to identify six additional areas sensitive to visual location. In the first step, regions were selected in each subject's left and right hemisphere based on the conservative multiple-comparisons corrected data from the polar-angle version of the delayed saccade task. If these maps revealed evidence for a region with a contralateral preference in the same general area, as defined by proximity to anatomical landmarks (see supplementary text S2), in more than half of the eight hemispheres examined, we used a less stringent statistical threshold to define the region in the remaining hemispheres. This criterion revealed evidence for six additional regions outside occipital cortex with a significant preference for the contralateral visual field. In the second step, we tested the reliability of these regions against data from the oddball task (see above). All six additional areas were significant using this random-effects test on an independent data set (Table 2).

**Figure 5:**
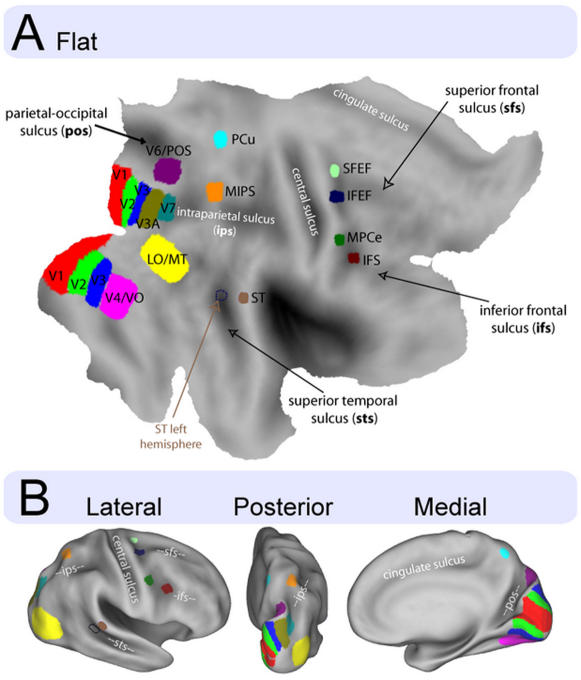
Schematic illustration of regions sensitive to visual field location on the PALS atlas. A shows a flat representation of the cortex of the right hemisphere. B shows three different views of an inflated representation of the same surface. PCu, precuneus; ST, superior temporal; SFEF, superior FEF; IFEF, inferior FEF; MPCe, middle precentral; IFS, inferior frontal sulcus. The atlas co-ordinates of these regions are listed in Table 2.

The six additional regions were named based on their anatomical proximity to gyral or sulcal landmarks, with the exception of the FEF region, which was already defined in prior work [35]: PCu, precuneus; ST, superior temporal; SFEF, superior FEF; IFEF, inferior FEF; MPCe, middle precentral; IFS, inferior frontal sulcus. They are shown schematically in Figure 5. The atlas co-ordinates of these regions are listed in Table 2 and their location relative to anatomical landmarks are described in Supplementary text S2. Figure S3 shows cortical trajectory analyses of these six extra-occipital regions, illustrating the absence of any clear topographic organization. All but one of these regions, ST (Superior Temporal), has also been observed in phase encoding studies [12].

### Region-based quantitative assessment of organization

The results of the cortical trajectory analyses might depend on the exact cortical trajectory chosen (but see figure S4). To overcome this concern, and to provide a more thorough quantitative assessment of visual field organization across areas, we devised a separate region based analysis (see methods). This analysis is also significant since it controls for effects of receptive field size.

**Figure 6:**
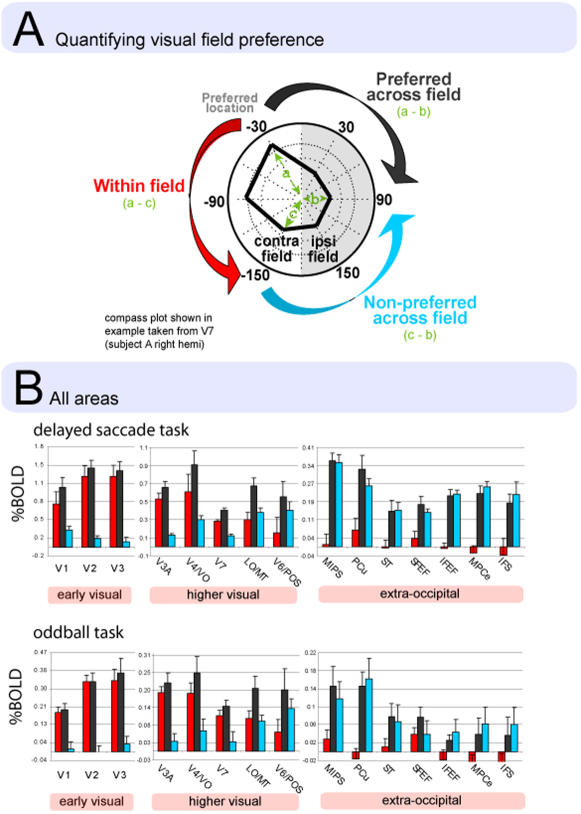
Quantification of visual field organization. A shows how the measures are calculated. The analysis was restricted to voxels which preferred either the upper or lower contralateral visual field locations. Each measure involves a subtraction of BOLD magnitudes associated with two visual field positions. The measures are comparable in the sense that the visual distance between locations was the same for each comparison. Importantly, increases in receptive field size should influence all measures equally. The black bars show the difference in BOLD response between the preferred visual field location and an ipsilateral location. Voxels were selected on the basis of contralateral preference, and so this measure can be considered as a baseline for comparison. The red bars reflect the difference between preferred and non-preferred within field locations. This measure reflects the degree of topography present in the region. In early visual areas, the red and black bars are identical, indicating a degree of topographic specificity as great as the contralateral preference. The blue bars indicate the difference between the non-preferred visual field location and the ipsilateral location. This measure reflects the degree to which the region possesses a non-topographic preference for the contra-lateral field. In extra-occipital areas, the black and blue bars are identical, indicating that these regions demonstrate a preference for the contra-lateral visual field but no detectable topography. Higher visual areas demonstrate a transition between these two types of organization.

An area that shows a largely contralateral organization should demonstrate two related characteristics. First, responses should change much more strongly to fixed changes in polar angle that cross the vertical meridian than to changes that do not. For example, Figure 6A illustrates a voxel that responds preferentially to a visual target at −30 deg as opposed to the other five locations. If that preference mainly reflects a contralateral bias, then responses should be much weaker to a location that is 120 deg distant in polar angle and crosses the vertical meridian (i.e. the location at 90 deg, shown by the black arrow) than to a location that is 120 deg distant but remains within the contralateral visual field (i.e. the location at −150 deg, shown by the red arrow). In Figure 6B, the black bars plot the difference score when the vertical meridian is crossed (−30 vs 90), the red bars when the meridian is not crossed (−30 vs −150). In extra-occipital areas (e.g. MIPS) the scores were much larger when the vertical meridian was crossed, while in occipital areas (e.g. V1) the two scores were much more equivalent, reflecting the fact that the relative BOLD responses to two locations in these areas were much less affected by whether the meridian was crossed. These results were highly consistent over the two tasks.

A corollary of the first property of contralateral organization is that any contralateral location should yield a larger response than any ipsilateral location. A 120 deg change in polar angle that crosses the vertical meridian should yield a similar change in response whether the starting point is the preferred contralateral location (black arrow in Figure 6A) or a non-preferred contralateral location (blue arrow in Figure 6A). Figure 6B shows that responses in extra-occipital areas changed equivalently for preferred (black bars) and non-preferred (blue bars) starting points. Conversely, in early visual areas preferred starting points (black bars) yielded much larger changes in BOLD response than non-preferred starting points (blue bars). These results were highly consistent over the two tasks.

Therefore, both measures of contralateral dominance indicated low dominance in early visual areas (red = black≫blue) and high dominance in extra-occipital areas (red≪black = blue). The predominant functional organization of visual responses in extra-occipital areas to be a broad selectivity for the contralateral visual field with a significant response to ipsilateral stimuli (about half of the contralateral response, PCu excepted). Topographic signals may be present but represent a small fraction of the total signal measured in these areas.

In addition to this region-based analysis, we computed separate normalized measures of contralateral preference and topography (see figure S3). These findings were highly consistent with those shown in Figure 6.

### Eccentricity organization

**Figure 7:**
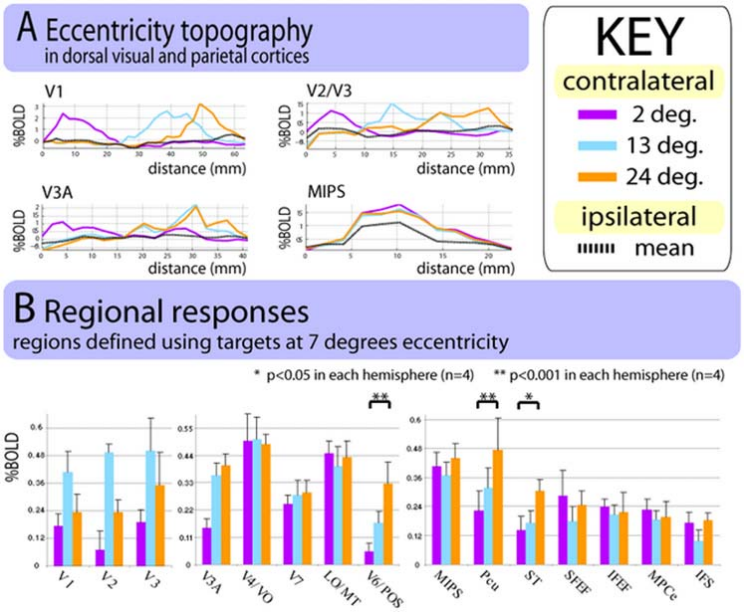
Results from the eccentricity version of the delayed saccade task, in which targets lay at one of three eccentricities (2, 13 and 24 degrees visual angle) on the horizontal meridian. A illustrates topographic organization in dorsal visual and parietal areas. The graphs plot the magnitude of BOLD response to three locations of varying eccentricity in the contralateral visual field, by distance across the cortical surface. The dotted black lines show the mean response to ipsilateral locations. Cortical trajectories were drawn using data from the passive retinotopy task. The trajectories for V1, V2/V3 and V3A each followed representations of the horizontal meridian in dorsal occipital cortex (these are the solid lines shown in Figure 3). The trajectory for MIPS was drawn at the same orientation on the flat surface. B shows mean responses for regions. The regions were defined using data from the polar angle version of the delayed saccade task, in which the eccentricity of the targets was approx. 7 degrees visual angle. The bars plot the BOLD response to contra-lateral locations, with the mean response to ipsi-lateral targets subtracted. These values are averaged across hemispheres (n = 4), with error bars showing the standard error. Note that in early visual areas there is a clear preference for the middle (13 deg.) contralateral location. This location corresponded most closely to the location of targets used to define these regions, and thus this preference reflects the topographic organization of these areas. This effect is not present in intermediate and extra-occipital areas. However, a few areas (V6/POS, PCu, ST) showed a preference for more peripheral stimuli.

Up until this point, we have focused on polar-angle organization. In this final section, we examine the effect of varying the eccentricity of the target (Figure 7). The same progressive trends in visual field organization that were observed for polar angle responses were also observed for responses to stimuli at different eccentricities.

Figure 7A plots responses along cortical trajectories to targets at different eccentricities in the contralateral visual field using the eccentricity version of the delayed saccade task. The cortical trajectories for V1–V3A followed the representations of the horizontal meridian revealed by passive retinotopy (the solid lines shown in Figure 3). In V1–V3, the maximum response occurred in more eccentric locations as one moves along the cortical trajectory, reflecting the well-defined topography of these areas. In contrast MIPS showed similar responses to all eccentricities at all points along the cortical trajectory. Responses to the three eccentricities in the ipsilateral field were also highly similar (not shown). Therefore, MIPS was most reliably identified by a preference for the contralateral field, irrespective of whether stimulus location was varied by polar angle or by eccentricity (see also figure S5).

Figure 7B shows regional responses. The figure plots the responses to the 2, 13, and 24 deg eccentricity in previously defined regions. It is important to bear in mind that these regions were defined by activity in the polar angle task (see methods), in which the targets were at 7 degrees eccentricity. Areas with topographic eccentricity organization should show their largest response to the 13 degree target, since this is the closest to the reference eccentricity of 7 degrees. This is what was observed for areas V1–V3.

Areas lacking topography and in which all eccentricities are represented approximately equally should show approximately equal responses to the three eccentricities. This was observed for most regions, including MIPS.

Regions V6/POS, PCu, and ST demonstrated a graded preference for more eccentric visual field locations. This indicates that more peripheral visual areas are preferentially represented in these areas (although they do not appear to have any topographic organization, see also figure S4). We conducted regional contrasts (most eccentric minus least eccentric contralateral location) to assess the reliability of these preferences (see methods). For all three areas the effect was significant in each of the four hemispheres tested: V6/POS, p<0.001 for all cases (A left z = 17.1; A right z = 7.3; C left z = 9.1 ; C right z = 12.4). PCu, p<0.001 for all cases (A left z = 7.5; A right z = 3.4; C left z = 6.1; C right z = 9). ST, p<0.05 for all cases (A left z = 4.9; A right z = 4.75; C left z = 1.7; C right z = 4.8).

## Discussion

We investigated visual field organization in occipital and extra-occipital cortex by comparing BOLD responses to discrete stimulus locations. The results indicated progressive changes in visual field organization from early visual areas to extra-occipital areas. First, there was a tendency for spatial selectivity to decrease, as demonstrated by increases in the BOLD response associated with ipsilateral locations. Second, the spatial profile of the selective signal changed. Early visual areas showed a large difference in response to preferred compared with non-preferred visual locations, regardless of whether non-preferred locations lay within the contralateral or ipsilateral visual field. In contrast, extra-occipital areas showed a much more robust difference in response when non-preferred locations lay in the ipsilateral visual field as opposed to the contralateral visual field. Therefore, visual field organization showed a progressive change from topographic in early visual areas to contralateral in extra-occipital areas.

### Contralateral organization is more robust than topographic organization in extra-occipital areas sensitive to visual location

The current results indicate that as one moves from early visual areas to extra-occipital areas, topographic signals become difficult to detect while contralateral preferences remain robust and extend throughout the entire contralateral field. These results suggest that extra-occipital areas whose response depends on visual location are more reliably determined by measuring contralateral preference than by searching for a complete, ordered phase representation of polar angle. The present results are not inconsistent with the presence of topography in previous phase-encoding studies of extra-occipital cortex, since our study may have had lower signal-to-noise, for instance because we used a head coil [but see 12 who also used a head coil for some subjects]. Nonetheless, any absolute difference in sensitivity does not bear on the progressive changes in visual field organization noted above (i.e. multiplying all responses by a factor of two would not affect the relative changes in field organization documented in Figure 6).

### The organization of regions sensitive to visual location in the intraparietal sulcus

The current literature on this topic is unsettled since the two principal groups that have studied IPS using phase encoding methods have reported divergent results. Sereno et al. [10] reported a topographic region in medial IPS that (as suggested by Silver et al. [11]) may lie anterior to two IPS regions, IPS1 and IPS2, discovered by Schluppeck et al. [9] and Silver et al. [11]. These latter two regions extended from V7, indicating a continuous tiling along an axis of occipital-parietal cortex. In contrast, the area of Sereno et al was isolated from the retinotopic belt. It is unclear why Schluppeck et al. and Silver et al. did not find Sereno et al.'s area or why Sereno did not find the areas of Schluppeck et al. and Silver et al. The task used by Schluppeck et al was the same as that used by Sereno et al. A further issue is that Sereno et al reported that the orientation of the topographic map varied somewhat across subjects in IPS [10], as well as in other regions [12]. In contrast, Silver et al and Schluppeck et al report that IPS1 and IPS2 were oriented consistently across subjects, akin to the highly stereotyped organization seen for maps in early visual areas.

The present results show that these studies measured topographic signals which were either very small in magnitude, or entirely absent. Therefore, inconsistency of results across studies may not be surprising. Figure S6 (see also discussion in supplementary text S1) shows that there was little evidence of either contralateral preference or of topographic organization in the area separating V7 and MIPS.

The current results in IPS appear most consistent with those of Sereno et al. since: (i) area MIPS was clearly separated from V7 (ii) the location relative to anatomical landmarks (see supplementary text S2) and Talairach coordinates were broadly similar (MIPS lay approx 7 mm distance from Sereno et al's area, whose coordinates were 32, −64, 46 after MNI to Talaraich conversion [36]) (iii) in those subjects/hemispheres in which we found evidence of topographic maps, these were oriented in a manner consistent with the orientation described as predominant by Sereno et al [10].

It is possible that the areas identified by Schluppeck et al. and Silver et al. have contralateral preferences and topography that are too weak to be detected with the current methods/hardware. A second possibility is that MIPS matches IPS2. However, the Talairach coordinates for MIPS are considerably anterior to IPS2 (Silver et al report the average center of IPS2 as lying at 19, −75, 48, some 19 mm distance from the center of MIPS) and the topographic maps we observed for MIPS in 3/8 hemispheres had the reverse orientation to those reported for IPS2. A third, related possibility is that MIPS constitutes a part of IPS2, consistent with the pattern observed in the right hemisphere of subject D (Figure S2). It would be surprising if Schluppeck et al. and Silver et al. did not identify voxels in MIPS, since our results indicate that they can be detected more easily than voxels in IPS1 and IPS2. A fourth possibility is that, in some cases, regions on the medial surface have been inadvertently included in IPS1 and IPS2. While this does not appear to hold for Silver et al [11], close examination of Schluppeck et al [9], who use a task very similar to the delayed saccade task used here, appears to show this is true for half the cases examined (S1 left, S2 left, S2 right, S3 right). However, intraparietal sulcus can be distinguished from regions on the medial surface both on the basis of anatomical location and the tendency for medial regions to prefer more eccentric visual locations (see Pitzalis et al [37] and figure S6). A final possibility is that our method of combining bottom-up signals due to visual stimulation with top-down signals due to attention or eye-movement planning obscured responses in IPS1 and IPS2. However, Silver et al [11] showed modest but reliable activation of IPS1 and IPS2 in response to passive stimulation.

### Ipsilateral BOLD signals are present in extra-occipital areas sensitive to visual location

Most extra-occipital regions sensitive to visual location nevertheless showed substantial ipsilateral activations, roughly 50% of the signal evoked by contralateral stimuli (see figure S3). The one exception was PCu, which also showed a preference for very peripheral stimuli. Ipsilateral activations may reflect neurons within these areas that have very large receptive fields, i.e. many neurons in these regions may be insensitive to visual location, or neurons that have receptive fields centered in the ipsilateral visual field. In addition, the BOLD response may average signals in a neuron that occur at different times and show a different dependence on spatial location. In LIP, many neurons respond during the period immediately after a saccade has occurred, and this response has been found to be less spatially specific than earlier responses in the same neurons [38]. The current study mixed sensory and attentional signals, each of which may produce more or less ipsilateral activation. A final possibility is that ipsilateral BOLD activations reflect inhibitory inputs to these areas rather than excitatory neuronal responses. For example, if these regions are involved in selectively attending to a location and this process is implemented competitively between the two visual fields [e.g. as in biased competition, 39], they may receive inhibitory inputs from regions representing the other visual field. Inhibitory inputs are known to produce a positive BOLD response [30].

### Higher-order frontal, parietal, and temporal areas sensitive to visual location

In addition to MIPS, six extra-occipital regions showed signals that depended on visual location and demonstrated considerably more robust contralateral selectivity than topographic organization. Aside from PCu, each region also showed clear ipsilateral activations. Signals that depend on location have been previously reported in each region, with the exception of ST [10], [12]. Area ST on the posterior portion of the superior temporal gyrus matches a functional region active for reorienting attention to unattended visual targets (STG, 57,−45,12 vector distance = 7.2 mm; Corbetta, 2000; 2002), and for detecting salient sensory multimodal changes (54,−42,13 vector distance = 4.7 mm; Downar et al., 2000). Previous studies involving group-averaged data have failed to reveal contralateral organization. Interestingly, this region showed a preference for peripheral stimuli, consistent with its putative role in spatial re-orienting.

Area IFEF and SFEF are located at the intersection of superior frontal and precentral sulci, the putative human homologue of monkey FEF [35], [40]–[42]. The cortex that is responsive to visually guided eye movements consists of a strip of cortex that starts at the junction of superior frontal sulcus and precentral sulci (about z = 50–60 mm), moves laterally along the horizontal ramus of the precentral sulcus (from x = ±20 to x = ±40 mm), and extends ventrally along the ventral ramus of the precentral sulcus (from z = 50–60 mm to z = 30–40 mm). Petit and Beauchamp identified a dorsal precentral sulcus region that generally matches the location of SFEF and IFEF (Beauchamp: −26,−14,53 vector distance from left SFEF = 4.6 mm; 31,−8,52 vector distance from right SFEF = 13 mm; from right IFEF = 3.1 mm). Corbetta et al 1998 distinguished a dorsal precentral sulcus region (41,−7,46 vector distance from right IFEF = 13 mm; −35,−9,46 vector distance from left IFEF = 4.5 mm), and a precentral-superior frontal sulcus region (29,−3,60 vector distance from right SFEF = 11 mm; −27,−13,46 vector distance from left SFEF = 12.7 mm). These findings are consistent with the evidence of two dorsal frontal eye movement areas that show a robust contralateral preference.

Area MPCe corresponds to the ventral precentral eye movement region defined by Beauchamp et al (−44,−14,40 vector distance with left MPCe = 9.6 mm; 47,−6,40 vector distance with right MPCe = 2.2 mm). The IFS area was the only area that has not been reliably activated in studies of eye movements and attention, and has instead been associated with storage in working memory [43].

### Reliability of the results and possible artifacts

Because signals in extra-occipital areas that depend on visual location can be small in magnitude and quite variable, it is important to demonstrate the statistical significance of the results, not only within subjects, but also across subjects or hemispheres. The statistical significance within a subject of the contralateral preference of each of the 7 regions sensitive to visual location was established using the delayed-saccade data with voxel-level z-statistics that were multiple-comparison corrected over the entire brain. The statistical significance across subjects of the contralateral preference of each region defined by the delayed-saccade data was determined by conducting a regional random-effects group t-test on the data from the oddball task, an independent data set, with subjects/hemispheres as the random factor.

The across-tasks replication approach not only rigorously demonstrated the across-subject/hemisphere reliability of the results, but also eliminated potential explanations of the results that focused on the particular characteristics of one or the other tasks, since the two tasks involved different stimuli (randomly positioned dots vs, colored pictures), judgments (delayed saccade vs. identification of visual oddballs), baseline conditions (fixation vs. rapid serial presentation of standard objects), responses (eye movements vs. key-press) and attentional states (endogenous vs. stimulus-driven).

The reliability of the results demonstrated by the replication approach extended to the measures of visual field organization presented here (Figures 6 and S3). The two tasks produced highly similar estimates of visual field organization. All of the trends in that were evident with one task - e.g. in extra-occipital areas, the predominance of contralateral over topographic organization - were clearly evident with the other task.

The replication approach also answers a possible criticism of the current approach, namely, that since the voxels comprising each region were defined by contralateral preference, noise within those voxels biased the results toward greater contralateral preference than topography. However, this difference was demonstrated in a dataset that was separate from that used to define the regions, eliminating any bias in the noise.

In relation to the quantitative measures of topography used (Figures 6 and S3), there may a concern relating to the fact that we used one task to determine the preferred contralateral location in order to calculate an estimate of topography in the other task. The advantage of this approach is that it provides an unbiased method to estimate the preferred location and thus a true estimate of the degree of topographic organization. However, if it turned out that topographic organization was task dependent then this procedure would produce artificially reduced estimates of sensitivity to location within the contralateral field. This explanation of our findings is implausible for a number of reasons. First, there is no evidence of task dependent topography. Topographic organization clearly remains constant across tasks in occipital areas (e.g. as illustrated in Figure 3). Previous studies suggest that topographic organization also remains constant across tasks in extra-occipital areas [9]–[11]. Similarly, we found consistent topographic organization in one case for MIPS (figure S7). Second, it is unclear why contralateral organization should remain constant while topographic organization changes. Third, the qualitative analyses presented illustrate that contra-lateral selectivity was far more robust than topographic organization even when we look just at data from a single task, as illustrated in Figures 4, 7, S1, S2, S4, S5, S7.

Another concern is that the results were affected by the limited sampling of locations in each hemifield. In polar-angle scans of phase encoding procedures, the stimulus sweeps continuously through every location in the field. However, in V1–V3, the main effect of limited sampling in the ‘cortical-trajectory’ analyses of Figures 4 and 5 was to restrict the number of voxels that showed strong within-field indices, not to eliminate those voxels. In contrast, no voxels in extra-occipital areas showed strong or even moderate within-field indices. Increasing the number of spatial samples would not have changed this result.

A final methodological concern is that the results may be partly due to the limited spatial resolution of fMRI, and in particular to partial volume averaging. Data relevant to this issue is presented in supplementary text S1 and figure S7. It is likely that partial volume effects influence our results to some degree. Nonetheless, as discussed in the supplementary text S1, the progressive changes in organization observed cannot be easily explained by this factor alone.

### Comparison with monkey electrophysiology

The progressive changes in human topography observed here are similar to that observed in the macaque using electrophysiological methods. Felleman and Van Essen (1991) distinguish four categories of topographic organization in the monkey. The first visual areas, V1, V2, V3 and VP all fall into the first two categories, with well defined topography. Similarly we find a high within-field index of at least 70% for these areas in the human (see figure S6). This means that BOLD response to non-preferred locations within the contralateral field was 70% smaller than the response to the preferred location, on average. Felleman and Van Essen (1991) describe the topography of more anterior areas on the ventral, lateral and dorsal occipital surfaces, including V3A, V4 and MT as coarse and irregular. Correspondingly we measure a more modest within-field index of 20–50% for these areas in the human (50% for V3A, 30% for V4/VO, and 20% for MT/LO).

Very crude topography has been observed in monkey LIP [4], corresponding to the low within-field value of ∼10% observed in human MIPS. The same single-unit study of monkey LIP found that, while some neurons preferred the ipsilateral visual field, the majority of neurons preferred the contralateral visual field. Again these findings are in accord with the current results for MIPS [but see 21 who did not see a tendency for neurons to prefer the contralateral field].

These data indicate that as one moves from early to higher visual areas in the human, just as in the monkey, contralateral preference remains clearly identifiable while topographic signals become difficult to detect.

However, a detailed comparison of monkey and human maps will require measurements involving the same technique, since single-unit recording and BOLD imaging differ along many dimensions. For example, single unit recordings involve post-hoc reconstructions of the position of receptive fields where sampling biases and inaccuracies in reconstruction may be present. Interestingly, two recent studies of monkey parietal cortex using optical imaging, which allows a bird's eye view of an entire area, have found some evidence of topography. However, the mapped variable was eye position rather than retinal location (Raffi and Siegel, 2001; 2002), again consistent with the current results. Another difference is that BOLD signals may predominately reflect afferent inputs to areas, rather than the firing of neurons in those areas [30].

A recent inactivation study reports evidence of well defined topographic organization in macaque principal sulcus, as assessed by deficits in performance [6]. It is possible that a small patch of cortex whose inputs and/or neurons represent locations across the visual field nonetheless has outputs and/or a functional role specific to a particular portion of the visual field. There is also evidence of topographic organization by eccentricity in monkey frontal eye fields, as assessed by the amplitude of saccades elicited by microstimulation [44]. We looked for evidence of similar organization in human frontal cortex, however any such organization was too weak for us to detect. We can not rule out the presence of crude eccentricity topography in this area, however the topography revealed by microstimulation in the monkey appears to be well defined. One possibility is that these eccentricity maps would be evident during saccade execution. The delayed saccade task used here and in previous studies [9], [10] has limited ability to detect activity associated with the act of making a saccade, since saccades occur infrequently and it is not possible to separate out the BOLD response associated with outbound and return saccades. Rather, the most significant aspect of this task, at least in relation to visual field dependent responses in parietal cortex, appears to be the need to remember the intended saccade location during the delay period (see [10], [18], [19], [33], [45]). An alternative possibility, similar to our discussion of principal sulcus above, is that there is topographic organization of outputs from FEF, but not of inputs. There is evidence of eccentricity topography in projections from macaque FEF to visual areas & LIP [46], and superior colliculus [47]. Finally, there is always a possibility that these areas are not homologous between macaque and human.

### Methodological significance for phase-encoding studies

Phase-encoding studies aim to reveal topographic organization in order to identify the borders of distinct visual areas. Our findings support this method of identifying early visual areas. However, they also suggest caution when using phase-encoding methods to identify higher areas, particularly outside occipital cortex. The topographic signal is weaker in these areas, and thus may not prove reliable. Further, the finding that contra-lateral selectivity accounts for far more BOLD modulation than topographic organization raises specific methodological concerns for phase encoding. The typical approach in phase-encoding studies is to threshold phase maps using a statistical test that is sensitive to periodic activity at the frequency of the stimulus. However, contra-lateral organization alone will be sufficient to produce significant periodic activity. Thus, contrary to what is commonly assumed, the statistical test does not entail the presence of reliable topographic organization. The full effect of contralateral predominance on phase encoding measurements remains to be determined. Our findings raise a concern about the typical phase-encoding approach, and suggest that contralateral preference provides a more robust criterion than topography for identifying the borders of extra-occipital areas.

### The functional significance of contralateral organization

The changing nature of visual field organization may provide some insight into the function of these areas. The organization of brain areas into topographic maps is a recurring feature of early sensory areas, not just in vision but also in audition, touch and olfaction. Why do early visual areas possess a well defined topographic organization, and why does this organization change for higher visual areas?

A simple account might hold that the early visual areas merely preserve the spatial arrangement present in the retina, and that this organization becomes progressively more diluted at each synapse along the chain of processing. However, topographic maps need not match the spatial arrangement of incoming projections [48], and specific developmental mechanisms have been identified that aid the formation of topographic maps [for a review see 49].

The leading theoretical account is that topographic organization minimizes wiring costs for connecting neurons within an area that predominantly analyzes local spatial relationships [for a review see 31]. In early visual areas, neurons representing nearby visual locations need to combine information to aid the identification of visual features. An ordered retinotopic map minimizes wiring length by placing neurons with adjacent receptive fields as close to each other as possible. Chklovskii and Koulakov [31] suggest that the principle of wiring optimization is sufficiently robust to allow an inference from visual field organization to function: “If the representation of the visual field in that cortical area is…nonretinotopic, then the processing is not likely local in the visual space”.

Following Chklovskii and Koulakov's reasoning, the present findings may indicate that processing becomes progressively less local in visual space moving from early visual areas, through higher occipital areas, to extra-occipital regions. The processing in these latter regions may still be highly spatial, but not emphasize local spatial relationships over distant relationships. The absence of a local processing bias in these regions could be consistent with their involvement in saccade planning and/or the allocation of attention, since the selection of a single target location might reflect competition between neurons that represent all parts of the visual field.

However, this account does not explain why contralateral organization persists, even when topographic organization subsides. Contralateral organization might reflect a fundamental division between the left and right visual fields, such that processing within each field differs from processing that spans the vertical meridian. Individuals with damage to the corpus callosum show evidence of independent processing of attentional cues relevant to each hemisphere [50] and an increased cost for attentional shifts between visual fields [51]. These results indicate that compromised connectivity between neurons representing different visual fields does produce observable effects on attentional performance. However, since these effects were not observed in control groups with normal connectivity, neurons representing different visual fields appear just as well connected as neurons representing the same field, despite the expensive wiring costs of callosal connections. Rather than postulating a fundamental division in processing between visual fields, for which there is little evidence, a more parsimonious explanation for the predominance of contralateral over topographic organization is the need to minimize the wiring costs associated with connections between areas. Suppose, for example, that neurons in area X that represent the left visual field have a high degree of connectivity with other cortical areas in the right hemisphere, but few connections to other cortical areas in the left hemisphere. If these inter-area connections are sufficiently prevalent relative to intra-area connections, then locating these neurons in the right hemisphere would minimize wiring costs. Therefore, minimizing inter-area connection lengths would produce a contralateral organization for area X, even in the absence of a local processing tendency sufficient to create a robust topographic organization. Under this model, neurons in area X would have equal numbers of intra-area connections within and across hemispheres; however their inter-area connections would be strongly biased towards areas in the same hemisphere. Why should an ipsilateral bias occur for inter-area connections? One possibility is that neurons in area X are very strongly connected to neurons in early visual areas that represent the same location in visual space. For instance, they may output directly to early visual areas in order to modulate their activity. Alternatively, these neurons may be strongly connected to motor neurons that control effectors on the same side of space.

In conclusion, our finding of clear contralateral selectivity but weak or absent topography in extra-occipital areas may indicate that these areas are engaged in processing that is global across visual space and that these areas have strong direct connections to areas that are engaged in local processing. These features are consistent with a role for these areas in the control of spatial attention.

## Methods

### Subjects

Four healthy right-handed subjects (one female, ages 19–26) with normal vision were recruited. Informed consent was obtained according to procedures approved by the local human studies committee. Table 1 details subject participation.

### Apparatus

Stimuli for the delayed saccade task were generated using an Apple G4 Macintosh computer. A PC was used to generate stimuli and collect responses in the oddball task. In both cases the visual image was projected onto a screen at the head of the bore by a Sharp LCD projector. Subjects viewed the stimuli through a mirror attached to the head coil. Manual responses in the oddball task were obtained using an MRI-compatible fiber-optic keypad held in the right hand.

We did not record eye movements in the scanner. At the start of each scanning session, subjects practiced the task in the scanner control room, at which time the experimenter monitored the subject's eye-movements.

### Delayed saccade paradigm - polar angle version

All stimuli were bright white against a black background. Subjects fixated a central crosshair while a peripheral target location was briefly (0.5 sec) presented within one of six sectors (sectors were centered at 30, 90, 150, −30, −30, −150 polar degrees from the upper vertical meridian, and 6.75 degrees visual angle eccentricity). After target offset, fixation was maintained for a variable duration (1.5, 3 or 4.5 sec), during which random flickering dots were presented throughout the target sector (e.g., 15–45 degrees polar angle, 6–7.5 degrees eccentricity). A blank screen (0.7 sec) signaled the time for the saccade to the remembered target position, and back to the center. The fixation point then re-appeared. Trials occurred in blocks of three, with the three targets appearing at different locations within the sector (in random order at −5, 0, and +5 degrees polar angle from the sector center, and at a randomly determined eccentricity between 6 and 7.5 degrees). Within blocks, trials were separated by a 0.7 second fixation interval. Successive blocks were separated by a randomly selected variable fixation interval of 4, 6.5 or 9 secs. These prolonged and variable fixation intervals allowed estimation of the main effect of the task as compared to a fixation baseline. The delay durations for the three trials within each block were randomly selected from permutations that allowed for a fixed total delay length of 9 sec per block (25% of blocks had three delays of 3 sec, in the remaining blocks one of the six possible permutations of 1.5, 3 and 4.5 sec was selected at random). Sector location varied pseudo-randomly (counterbalanced within each scanner run) from block to block, with the constraint that sector side (left or right) alternated from one block to the next. The fixation-cross subtended 0.225 degrees visual angle, as did each of the square dots, which marked the target location and acted as delay-period distracters. During the delay period, on average slightly fewer than 6 dots were visible at any one time within a wedge of 30 degrees polar angle and between 6 and 7.5 degrees eccentricity. The screen was updated every 0.1 sec, at which time each dot had a 60% probability of being randomly re-generated. Overlapping and/or touching dots were avoided by setting dot locations discretely rather than continuously, such that there were 180 possible polar angles and 6 possible eccentricities, uniformly distributed.

### Delayed saccade paradigm - eccentricity version

In the eccentricity version of the delayed saccade paradigm the sector centers all lay on the horizontal meridian. There were three sectors per side, at 2, 13 and 24 degrees visual angle from fixation. The size of each sector and component stimuli was scaled linearly with distance from fixation, scaled to the same sizes used in the polar angle version. The wider field of view was achieved by using a larger mirror and a projector screen that was placed inside the bore of the scanner, much closer to the subject. Sector location was varied pseudo-randomly (counterbalanced within each scanner run) without any constraint on alternating side. In all other respects the design was identical to the polar angle version of the delayed saccade task.

### Oddball paradigm (polar angle only)

Stimuli were presented against a plain bright white background. A colored picture stimulus was presented twice during each fMRI frame (every 1+/−0.25 sec for subject A, every 1.25+/−0.25 sec for subjects B & D). On most trials the same ‘standard’ picture was presented at the fovea, while on ∼12% of trials a new picture was presented in one of 7 locations (fovea or 6 peripheral locations). All picture stimuli were presented for a duration of 0.15 sec. Each trial had an equal probability of being an oddball trial (p = 1/7), except that the trial after an oddball trial was always a standard trial. Each of the seven possible oddball target locations occurred with equal probability. A grey fixation diamond subtending 0.05 degrees visual angle was visible in the center of the screen at all times except when a central picture stimulus was present. Subjects were asked to maintain central fixation throughout. Subjects were required to respond to every picture stimulus, categorizing it either as ‘standard’ or ‘oddball’ using a key-press. Oddball stimuli were randomly selected with replacement from a pool of 100 pictures of everyday objects. Since approximately 500 oddball targets were presented in each scanner session, the same object was presented only 5 times over a session, out of a total of approximately 4000 stimulus presentations. The peripheral locations were centered at the same polar angles as for the delayed saccade paradigm, with stimuli occupying from 6.25 to 11.25 degrees visual angle from the fovea. Stimuli presented at the center subtended approx. 2.3 degrees visual angle.

### Image acquisition and pre-processing

An asymmetric spin-echo echoplanar imaging sequence was used to measure blood oxygenation-level-dependent (BOLD) contrast on a Siemens Allegra 3T scanner. In the delayed saccade paradigm 156 whole brain volume images were collected in each scanner run. In the oddball task there were 150 volumes per run. Either 39 or 40 contiguous 3.25 mm slices were acquired with 3.25×3.25 mm in-plane resolution, a slice TR of 0.0641 sec (volume TR of 2.5 or 2.564 sec), TE = 25, flip angle = 90°. Except for the images acquired for subject A in the oddball task, where 31 contiguous 4 mm slices were acquired, 4×4 mm in-plane resolution, a slice TR of 0.0645 sec (volume TR = 2 sec), TE = 25, flip angle = 90°. In the delayed saccade paradigm subjects A, B, C and D participated in 36, 36, 36 & 35 scanner runs of 156 consecutive volume images over three sessions. In the oddball task, subjects A, B & D participated in 44, 42 & 42 scanner runs of 150 consecutive volume images over three sessions. Realignment parameters for the functional data were calculated first within run, then across runs within a single session, then across sessions. The functional data was re-sampled once directly to atlas space with a uniform voxel size of 3 mm^3^. The strategy of using a single re-sampling of the functional data to simultaneously correct for head movement within and across runs as well as effect an atlas transformation was found in previous comparisons to substantially improve signal to noise and reduce smoothing due to repeated resampling. For each peripheral location in the delayed saccade paradigm, subjects experienced ∼320 target location presentations of 0.5 sec, were required to remember those target locations while distracters were presented for ∼3 sec, and then made a rapid saccade to and from the target location. For each peripheral location in the oddball task, subjects experienced ∼200 stimulus presentations of 0.15 sec, and gave an immediate response to indicate that the standard stimulus, which always appeared in the center, had not occurred.

### Image analysis and statistics

Separate responses for each of the six peripheral locations (and a further response for the central oddball stimulus in the oddball task) were estimated at the voxel level using the general linear model. For the delayed saccade task, we assumed a sustained response over the three trials, modeled by convolving a gamma function with a delay of 2 sec and time constant of 1.25 sec (Boynton et al., 1996) with the duration of the block. In the oddball task we assumed an impulse response modeled using a gamma function. The design matrix was defined using impulse-basis functions such that at each frame, the data were modeled as the sum of the overlapping hemodynamic responses produced by each task effect plus a linear trend. Fixation periods in the delayed saccade paradigm and responses to ‘standard’ stimuli in the oddball task were not separately modeled but served as the baseline against which responses to peripheral stimuli were estimated.

To identify voxels with a contralateral preference, the response to sectors in the contralateral visual field minus the response to sectors in the ipsilateral visual field was first computed. The resulting t-statistic for each subject was converted to equally probable z-statistics prior to threshold and display. Statistical maps were corrected for multiple comparisons by thresholding at z>4 and only including clusters of 5 or more voxels. This z-score/cluster size criterion was conservative, corresponding to a multiple-comparison corrected threshold of p = 0.025.

For each subject, three anatomical MPRAGE images were averaged to produce a high-resolution structural image. Surefit and Caret (Van Essen et al., 2001) (http://brainmap.wustl.edu/caret) were used for surface generation & flattening, visual inspection, drawing & re-embedding of regions.

### Topographic maps and definition of regions

All regions, occipital and extra-occipital, used in analyses were defined on the basis of data from the polar angle version of the delayed saccade task, and comprised voxels that showed above threshold contralateral preference in that task (with the exception of IPS1&2, see below). For the purposes of delineating retinotopic areas, images were created in which each voxel was given one of three colors depending on which of the three contra-lateral stimulus positions produced the greatest BOLD response (Figure 3). Thus one color marked the horizontal meridian, one color indicated a 30 degree position from the upper vertical meridian, and the third color indicated 30 degrees from the lower vertical meridian. This is analogous to coloring voxels according to phase in the phase-encoding methodology. This image was thresholded by the whole brain multiple comparison corrected contrast contralateral minus ipsilateral. Regions V1, V2, V3, VP, V3A & V7 were drawn by reference to the established correspondences between their borders and the horizontal and vertical meridians. On the ventral occipital surface, we grouped visuotopic voxels anterior to the VP border as V4/VO, as we could not confidently separate V4 from other regions. Similarly on the lateral surface, in the absence of other localizers involving motion and/or presentation of specific objects, we could not confidently determine the location of different regions, and grouped all voxels showing evidence of lateralization as LO/MT. Voxels preferring the contralateral visual field which lay medial to V3/V3A/V7 were labeled V6/POS. These voxels primarily lay within the parieto-occipital sulcus, the described location of V6 (Pitzalis et al., 2006).

### Identification of extra-occipital areas

Because mapping studies are often based on a relatively small number of subjects in whom an extensive amount of data is collected, formal statistical tests of the reliability of results across subjects are not always provided. Because of the intrinsic anatomical variability of functional areas, a standard approach for assessing reliability across subjects is to qualitatively compare patterns of activation that are diagnostic of an area (e.g. polar angle topography) in approximately the same location or in relation to other functional markers (e.g. anterior to functionally defined area V7, Schluppeck et al., 2005; Silver et al., 2005).

In this study, we formally replicate the existence of extra-occipital areas showing a contralateral visual field preference using a two-step (hypothesis-test) approach, in which: 1) we selected a region (e.g. MIPS) with a significant contralateral preference in the delayed saccade task that showed a relatively consistent anatomical location across subjects and hemispheres, and 2) we replicated the contralateral preference of this area in an independent data set on the oddball task, including a random effects group statistic across hemispheres/subjects.

In the first step, the statistical map for the contrast contralateral minus ipsilateral, thresholded according to a conservative multiple-comparisons correction for the whole brain, was computed using data from the polar-angle version of the delayed saccade task. If these maps revealed evidence of a visual field-selective region in the same region, as defined by proximity to anatomical landmarks (see Supplementary text S2 for a description of the landmarks for each region), in more than half of the eight hemispheres examined, we used a less stringent statistical threshold to define the region in the remaining hemispheres. This criterion revealed evidence for seven regions (see Table 2 and Supplementary text S2) outside occipital cortex with a significant preference for the contralateral visual field. In the second step, we tested the reliability of these regions against data from the oddball task using two separate analyses. We computed for each individual the peak z-score for the contrast contralateral minus ipsilateral in the oddball task, i.e. oddball targets appearing in the contralateral vs. ipsilateral visual field, across all the voxels of the region, and then averaged this peak z-score over subjects and hemispheres. In addition, we performed a random effects analysis to establish that these a-priori areas were reliable across the population of hemispheres as a whole, and were not driven by one or two robust examples. We computed response magnitudes for each region for the horizontal contralateral and ipsilateral stimulus positions in the oddball task.

### Passive retinotopy

We collected passive retinotopy data for subjects A, B & C. Full field vertical and horizontal meridians, 4 Hz contrast reversing black and white checkerboards, were presented for 12.8 secs in alternating blocks; 10, 8 & 6 scanner runs for subjects A, B & C; 176 whole-brain volumes per scanner run; 40 contiguous 3.25 mm slices with 3.25×3.25 mm in-plane resolution, volume TR 2.564 sec). Rings were also presented at four different eccentricities (4 Hz contrast reversing black and white checkerboards, 12.8 second stimulus blocks alternating with 12.8 seconds fixation, random stimulus order; 4 scanner runs per subject, 145 whole brain volumes per scanner run, other parameters as for meridians). These data were used to verify the areal boundaries drawn on the basis of the delayed saccade data. The correspondence was excellent (see Figure 3). Passive presentation of horizontal and vertical meridians has previously been shown to produce results consistent with phase-encoding methods most commonly employed in investigations of visuotopic organization [27], [52].

### Graphs of BOLD magnitude along a cortical trajectory

An important tool for the qualitative assessment of visual field organization was the use of graphs that traced activity along a trajectory drawn on the cortical surface. These are shown in figures 4, 7, S1, S2, S4, S5, S7. These graphs were generated using Matlab programs written to operate on data files generated by Caret. Volume files were generated using the general linear model described above which provided estimates of the BOLD magnitude associated with discrete visual locations. These images then were projected on to the surface using Caret, a process that involves assigning a magnitude to each surface node depending on the value of the voxel in which the node lies in volume space. A transformation is calculated by virtually cutting and warping the cortical surface so that each node has defined coordinates both in talairach volume space and in a flat (two dimensional) coordinate system. Cortical trajectories are drawn by hand in the flat coordinate system, and consist of a series of points spaced approximately 2 mm apart. While distances on the flat surface are prone to distortion due to the warping that occurs as part of the flattening process, they are normalized over the whole surface so that the total cortical surface area is preserved. The magnitude of BOLD response at each point along the trajectory was established by taking the mean BOLD magnitude associated with every node which (i) lay within 2 mm of that point on the flat surface (ii) was closer to that point than to neighboring points on the trajectory.

### Quantitative measures of visual field organization

An important goal of this experiment was to extend our qualitative examinations of visual field organization by quantifying visual field organization. The measures of within field, preferred across field and non-preferred across field BOLD modulation shown in Figure 6 were calculated as follows. For the delayed saccade (oddball) task estimates, the preferred polar angle for each voxel was calculated using data from the oddball (delayed saccade) task data. This ensured an unbiased estimate of preferred location. Only voxels that preferred either the upper or lower contra-lateral visual field positions were included. Each measure was calculated separately for each voxel, as described in Figure 6. An estimate of each measure was calculated separately for each hemisphere/subject and for each region by averaging across all included voxels. The graphs show the mean and standard error of these estimates over subjects/hemispheres.

The lateralization and within-field indices shown in figure S3 provided estimates of contra-lateral preference and topographic modulation that were normalized across areas, making it easier to compare areas. The lateralization index was calculated using the formula: Lateralization index = (contra−ipsi)/contra. The within field index was calculated using the formula: Within-field index = (preferred−mean (non-preferred))/preferred. The preferred location used to calculate the within field index for the delayed saccade task was established using data from the oddball task, and vice-versa. In each case, the normalization was accomplished by dividing by the BOLD response associated with the preferred visual field location. However, when this denominator is small it leads to unstable estimates. To compensate for this problem, we calculated the mean value associated with numerator and denominator separately for each subject/hemisphere by averaging those values over all voxels with above baseline responses to the preferred location(s). The value of the index was then calculated for each subject/hemisphere using these corrected mean values. Graphs show the mean and standard error of these estimates across subjects/hemispheres. Including voxels in which the BOLD response to the preferred location(s) was below zero produced a qualitatively similar but noisier pattern.

## Supporting Information

Figure S1 Visual field organization of dorsal visual areas and medial intra-parietal sulcus - data for left and right hemispheres of subjects A and B. See figure 4 for details. The lower two panels quantify the degree of topographic organization (within max) and contralateral preference (laterality), allowing the reader to visualize the relative magnitude of these features as we move from early visual to parietal cortex. They are described further in the supplementary text.
(6.28 MB TIF)

Figure S2 Visual field organization of dorsal visual areas and medial intra-parietal sulcus - data for left and right hemispheres of subjects C and D. See figure 4 and supplementary text for details.
(5.67 MB TIF)

Figure S3 Lateralization and within-field indices for both tasks. Both indices measure the change in BOLD response magnitude due to variation in retinotopic stimulus location, derived from data from the polar angle version of the delayed saccade task and the oddball task. The lateralization index compares the mean response to contra-lateral visual locations with the mean response ipsi-lateral visual locations. The within-field index compares upper, middle and lower field targets in the preferred (contra-lateral) visual field. The measures are based on the BOLD response observed to a target in the preferred location, and indicate the average percentage decrease in BOLD response for targets in non-preferred locations (see methods). Error bars show standard error of the mean value, computed across hemispheres. V4/VO - voxels preferring the contralateral field anterior to VP on the ventral surface. LO/MT - voxels preferring the contralateral field anterior to the foveal confluence and early visual areas on the lateral surface. V6/POS - voxels preferring the contralateral field medial to V3A/V7, primarily located within the parietal-occipital sulcus.
(0.26 MB DOC)

Figure S4 Visual field organization of other extra-occipital regions. We took the most robust example of each region from the two subjects (A and C) who participated in both polar-angle and eccentricity versions of the delayed saccade. We drew two trajectories through each region, and plotted BOLD activity corresponding the three contra-lateral locations in the polar angle (top graph) and eccentricity (middle graph), with mean BOLD response to ipsi-lateral shown by a dotted black line. The bottom of the three graphs for each area shows the mean difference between contralateral and ipsilateral positions for the two data sets (polar angle and eccentricity), with the scale normalized for comparison.
(9.28 MB TIF)

Figure S5 Profiles of activity for cortical trajectories cross-secting area MIPS at three different orientations. An inflated representation of the left hemisphere of subject A is shown, overlayed with a statistical map showing voxels that prefer the contralateral visual field. Trajectories were drawn through area MIPS at three different orientations. The graphs below show the profile of activity along the trajectories, labeled 1–3. (A) shows data from the polar angle version of the delayed saccade task. The three contra-lateral locations are color coded as shown in the key. The dotted black line shows the mean activity due to ipsi-lateral locations. (B) shows data from the oddball task, displayed in the same format (C) shows data from the eccentricity version of the delayed saccade task, with contralateral locations color coded as shown in the key. (D) shows the mean difference between contralateral and ipsilateral field locations for the three tasks, with the scale normalized for comparison. Note that there is evidence of polar angle topography along trajectory (1), with the lower field represented more anterior and the upper field more posterior. This topographic organization was consistent across the two tasks. Nonetheless, BOLD modulation associated with topography was slight compared with the contralateral preference seen for this area. Note the highly consistent profile of contralateral preference for the three tasks illustrated in (D). The dotted circle in the top figure shows the location of area ST in the left hemisphere.
(7.40 MB TIF)

Figure S6 Visuotopic organization of intraparietal sulcus and surrounding cortex. A shows the abrupt change in the degree of topographic organization that occurs between V7 to MIPS. See Figure 6 for further explanation of the graphs. Previous studies have claimed a continuous retinotopic organization stretching along intraparietal sulcus. However the cortical area seperating V7 from MIPS, indicated here as IPS1/2, shows little evidence of contralateral preference or of topographic organization. B illustrates eccentricity organization in intraparietal sulcus and more medial regions. See figure 7 for further explanation of graphs. Regions within intraparietal cortex can be clearly distinguished from more medial regions on the basis of eccentricity preference.
(0.86 MB TIF)

Figure S7 Comparison of best example of MIPS topography (Subject C, right hemisphere) with area V3A in the same hemisphere. The figure illustrates that the reduced topographic organization of area MIPS cannot be accounted for by partial volume effects or by noise-induced spatial smoothing. A shows an inflated representation of the cortical surface with trajectories drawn to optimally capture topographic organization in MIPS and V3A. In area MIPS there is a high degree of correlation between the three contralateral locations. The topography in area V3A is much more clearly defined. The difference between the two areas cannot be attributed to distance, as illustrated by the x-axis of the graphs. In B the graphs trace 5 face-connected voxels that follow the cortical surface (indicated by a white line). Again, response profiles for different visual field positions are highly correlated in MIPS and clearly dissociate in area V3A. The contrast between areas is even more striking in the majority of cases, in which MIPS had no discernable topography.
(6.56 MB TIF)

Supplementary Text S1 (0.05 MB DOC)

Supplementary Text S2 (0.03 MB DOC)

## References

[1] DeYoe EA, Carman GJ, Bandettini P, Glickman S, Wieser J (1996). Mapping striate and extrastriate visual areas in human cerebral cortex.. Proc Natl Acad Sci U S A.

[2] Sereno MI, Dale AM, Reppas JB, Kwong KK, Belliveau JW (1995). Borders of multiple visual areas in humans revealed by functional magnetic resonance imaging.. Science.

[3] Engel SA, Glover GH, Wandell BA (1997). Retinotopic organization in human visual cortex and the spatial precision of functional MRI.. Cereb Cortex.

[4] Ben Hamed S, Duhamel JR, Bremmer F, Graf W (2001). Representation of the visual field in the lateral intraparietal area of macaque monkeys: a quantitative receptive field analysis.. Exp Brain Res.

[5] Constantinidis C, Franowicz MN, Goldman-Rakic PS (2001). Coding specificity in cortical microcircuits: a multiple-electrode analysis of primate prefrontal cortex.. J Neurosci.

[6] Sawaguchi T, Iba M (2001). Prefrontal cortical representation of visuospatial working memory in monkeys examined by local inactivation with muscimol.. J Neurophysiol.

[7] Suzuki H, Azuma M (1983). Topographic studies on visual neurons in the dorsolateral prefrontal cortex of the monkey.. Exp Brain Res.

[8] Blatt GJ, Andersen RA, Stoner GR (1990). Visual receptive field organization and cortico-cortical connections of the lateral intraparietal area (area LIP) in the macaque.. J Comp Neurol.

[9] Schluppeck D, Glimcher P, Heeger DJ (2005). Topographic organization for delayed saccades in human posterior parietal cortex.. J Neurophysiol.

[10] Sereno MI, Pitzalis S, Martinez A (2001). Mapping of contralateral space in retinotopic coordinates by a parietal cortical area in humans.. Science.

[11] Silver MA, Ress D, Heeger DJ (2005). Topographic maps of visual spatial attention in human parietal cortex.. J Neurophysiol.

[12] Hagler DJ, Sereno MI (2006). Spatial maps in frontal and prefrontal cortex.. Neuroimage.

[13] Yantis S, Schwarzbach J, Serences JT, Carlson RL, Steinmetz MA (2002). Transient neural activity in human parietal cortex during spatial attention shifts.. Nat Neurosci.

[14] Koyama M, Hasegawa I, Osada T, Adachi Y, Nakahara K (2004). Functional magnetic resonance imaging of macaque monkeys performing visually guided saccade tasks: comparison of cortical eye fields with humans.. Neuron.

[15] Macaluso E, Driver J, Frith CD (2003). Multimodal spatial representations engaged in human parietal cortex during both saccadic and manual spatial orienting.. Curr Biol.

[16] Macaluso E, Frith CD, Driver J (2002). Directing attention to locations and to sensory modalities: multiple levels of selective processing revealed with PET.. Cereb Cortex.

[17] Claeys KG, Lindsey DT, De Schutter E, Orban GA (2003). A higher order motion region in human inferior parietal lobule: evidence from fMRI.. Neuron.

[18] Medendorp WP, Goltz HC, Vilis T, Crawford JD (2003). Gaze-centered updating of visual space in human parietal cortex.. J Neurosci.

[19] Merriam EP, Genovese CR, Colby CL (2003). Spatial updating in human parietal cortex.. Neuron.

[20] Felleman DJ, Van Essen DC (1991). Distributed hierarchical processing in the primate cerebral cortex.. Cereb Cortex.

[21] Platt ML, Glimcher PW (1998). Response fields of intraparietal neurons quantified with multiple saccadic targets.. Exp Brain Res.

[22] Tootell RB, Mendola JD, Hadjikhani NK, Ledden PJ, Liu AK (1997). Functional analysis of V3A and related areas in human visual cortex.. J Neurosci.

[23] Hadjikhani N, Liu AK, Dale AM, Cavanagh P, Tootell RB (1998). Retinotopy and color sensitivity in human visual cortical area V8.. Nat Neurosci.

[24] Brewer AA, Liu J, Wade AR, Wandell BA (2005). Visual field maps and stimulus selectivity in human ventral occipital cortex.. Nat Neurosci.

[25] Wade AR, Brewer AA, Rieger JW, Wandell BA (2002). Functional measurements of human ventral occipital cortex: retinotopy and colour.. Philos Trans R Soc Lond B Biol Sci.

[26] Huk AC, Dougherty RF, Heeger DJ (2002). Retinotopy and functional subdivision of human areas MT and MST.. J Neurosci.

[27] Tootell RB, Mendola JD, Hadjikhani NK, Liu AK, Dale AM (1998). The representation of the ipsilateral visual field in human cerebral cortex.. Proc Natl Acad Sci U S A.

[28] Kraft A, Schira MM, Hagendorf H, Schmidt S, Olma M (2005). fMRI localizer technique: efficient acquisition and functional properties of single retinotopic positions in the human visual cortex.. Neuroimage.

[29] Hasson U, Levy I, Behrmann M, Hendler T, Malach R (2002). Eccentricity bias as an organizing principle for human high-order object areas.. Neuron.

[30] Logothetis NK, Wandell BA (2004). Interpreting the BOLD signal.. Annu Rev Physiol.

[31] Chklovskii DB, Koulakov AA (2004). Maps in the brain: what can we learn from them?. Annu Rev Neurosci.

[32] Corbetta M, Shulman GL (2002). Control of goal-directed and stimulus-driven attention in the brain.. Nat Rev Neurosci.

[33] Schluppeck D, Curtis CE, Glimcher PW, Heeger DJ (2006). Sustained activity in topographic areas of human posterior parietal cortex during memory-guided saccades.. J Neurosci.

[34] Tootell RB, Hadjikhani N, Hall EK, Marrett S, Vanduffel W (1998). The retinotopy of visual spatial attention.. Neuron.

[35] Paus T (1996). Location and function of the human frontal eye-field: a selective review.. Neuropsychologia.

[36] Brett M, Johnsrude IS, Owen AM (2002). The problem of functional localization in the human brain.. Nat Rev Neurosci.

[37] Pitzalis S, Galletti C, Huang RS, Patria F, Committeri G (2006). Wide-field retinotopy defines human cortical visual area v6.. J Neurosci.

[38] Barash S, Bracewell RM, Fogassi L, Gnadt JW, Andersen RA (1991). Saccade-related activity in the lateral intraparietal area. I. Temporal properties; comparison with area 7a.. J Neurophysiol.

[39] Desimone R, Duncan J (1995). Neural mechanisms of selective visual attention.. Annu Rev Neurosci.

[40] Corbetta M, Akbudak E, Conturo TE, Snyder AZ, Ollinger JM (1998). A common network of functional areas for attention and eye movements.. Neuron.

[41] Petit L, Clark VP, Ingeholm J, Haxby JV (1997). Dissociation of saccade-related and pursuit-related activation in human frontal eye fields as revealed by fMRI.. J Neurophysiol.

[42] Beauchamp MS, Petit L, Ellmore TM, Ingeholm J, Haxby JV (2001). A parametric fMRI study of overt and covert shifts of visuospatial attention.. Neuroimage.

[43] Smith EE, Jonides J (1999). Storage and executive processes in the frontal lobes.. Science.

[44] Bruce CJ, Goldberg ME, Bushnell MC, Stanton GB (1985). Primate frontal eye fields. II. Physiological and anatomical correlates of electrically evoked eye movements.. J Neurophysiol.

[45] Medendorp WP, Goltz HC, Vilis T (2006). Directional selectivity of BOLD activity in human posterior parietal cortex for memory-guided double-step saccades.. J Neurophysiol.

[46] Stanton GB, Bruce CJ, Goldberg ME (1995). Topography of projections to posterior cortical areas from the macaque frontal eye fields.. J Comp Neurol.

[47] Sommer MA, Wurtz RH (2000). Composition and topographic organization of signals sent from the frontal eye field to the superior colliculus.. J Neurophysiol.

[48] Ressler KJ, Sullivan SL, Buck LB (1993). A zonal organization of odorant receptor gene expression in the olfactory epithelium.. Cell.

[49] McLaughlin T, O'Leary DD (2005). Molecular gradients and development of retinotopic maps.. Annu Rev Neurosci.

[50] Arguin M, Lassonde M, Quattrini A, Del Pesce M, Foschi N (2000). Divided visuo-spatial attention systems with total and anterior callosotomy.. Neuropsychologia.

[51] Hines RJ, Paul LK, Brown WS (2002). Spatial attention in agenesis of the corpus callosum: shifting attention between visual fields.. Neuropsychologia.

[52] Fize D, Vanduffel W, Nelissen K, Denys K, Chef d'Hotel C (2003). The retinotopic organization of primate dorsal V4 and surrounding areas: A functional magnetic resonance imaging study in awake monkeys.. J Neurosci.

