# Supplementary figures and images for: Changing Human Visual Field Organization from Early Visual to Extra-Occipital Cortex
Source: PLoS One. 2007 May 16;2(5):e452. doi: 10.1371/journal.pone.0000452 (PMC1866221; doi:10.1371/journal.pone.0000452)

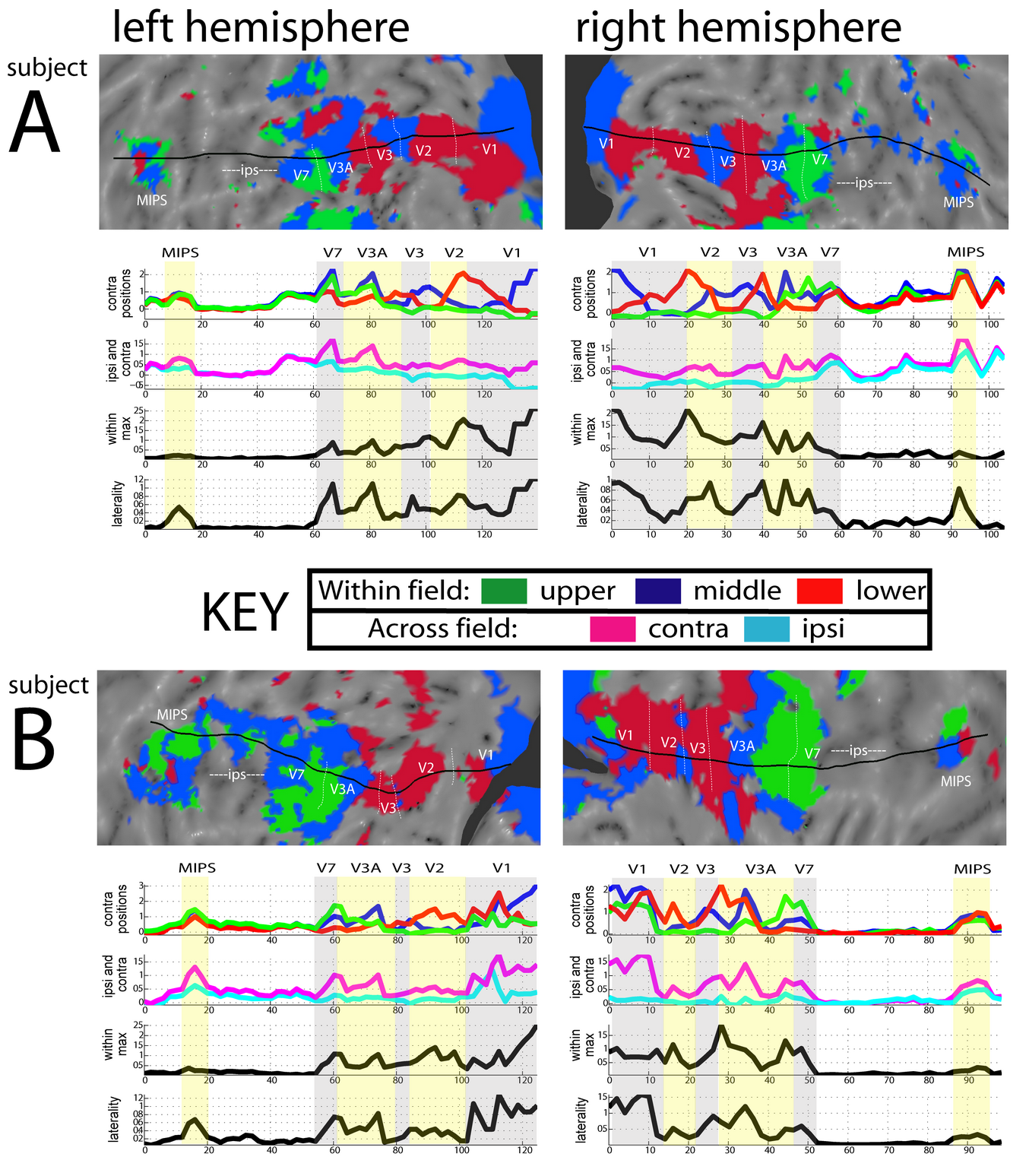

Supplement: Figure S1 — Visual field organization of dorsal visual areas and medial intra-parietal sulcus - data for left and right hemispheres of subjects A and B. See figure 4 for details. The lower two panels quantify the degree of topographic organization (within max) and contralateral preference (laterality), allowing the reader to visualize the relative magnitude of these features as we move from early visual to parietal cortex. They are described further in the supplementary text. (6.28 MB TIF) [file pone.0000452.s001.tif]

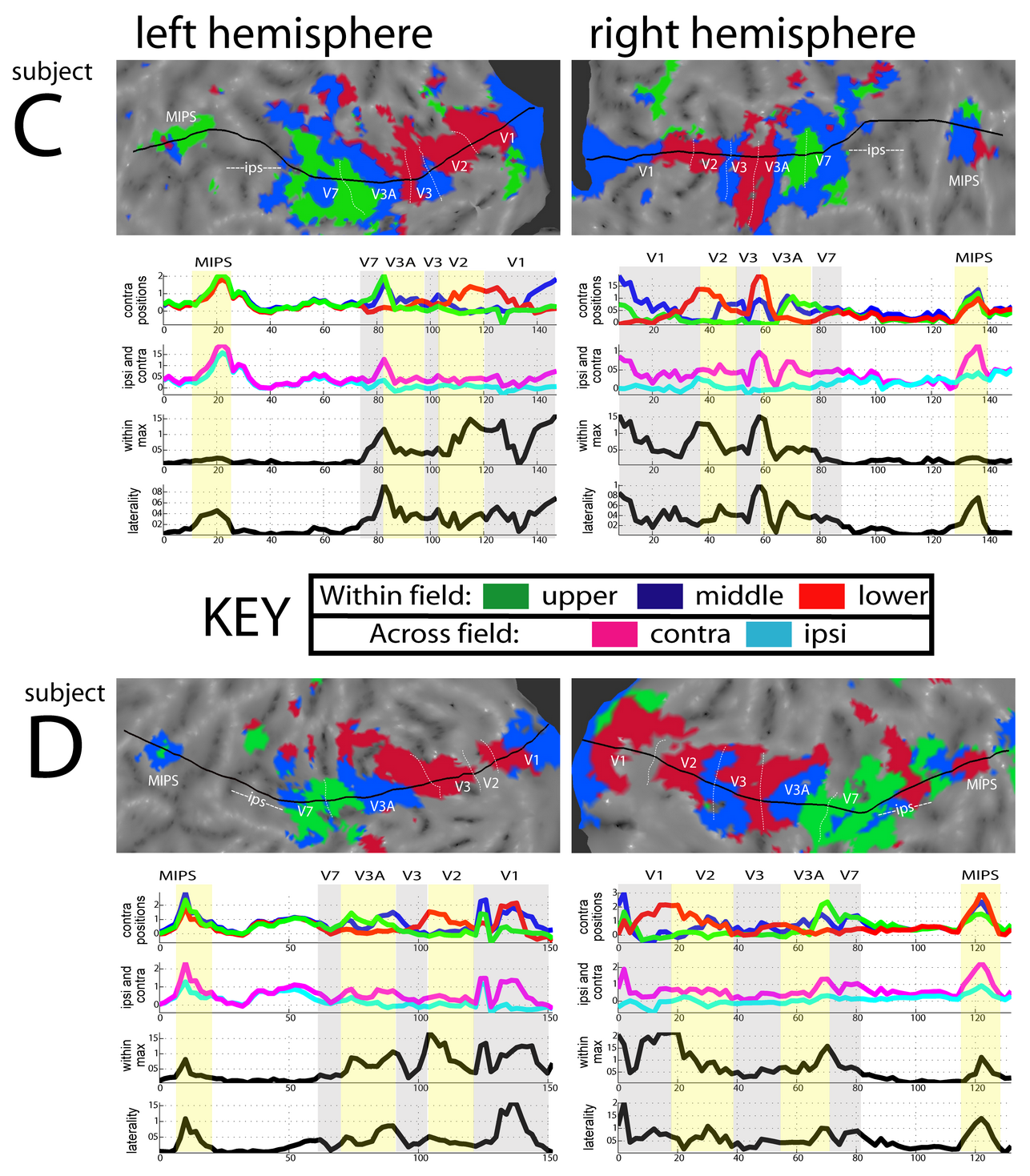

Supplement: Figure S2 — Visual field organization of dorsal visual areas and medial intra-parietal sulcus - data for left and right hemispheres of subjects C and D. See figure 4 and supplementary text for details. (5.67 MB TIF) [file pone.0000452.s002.tif]

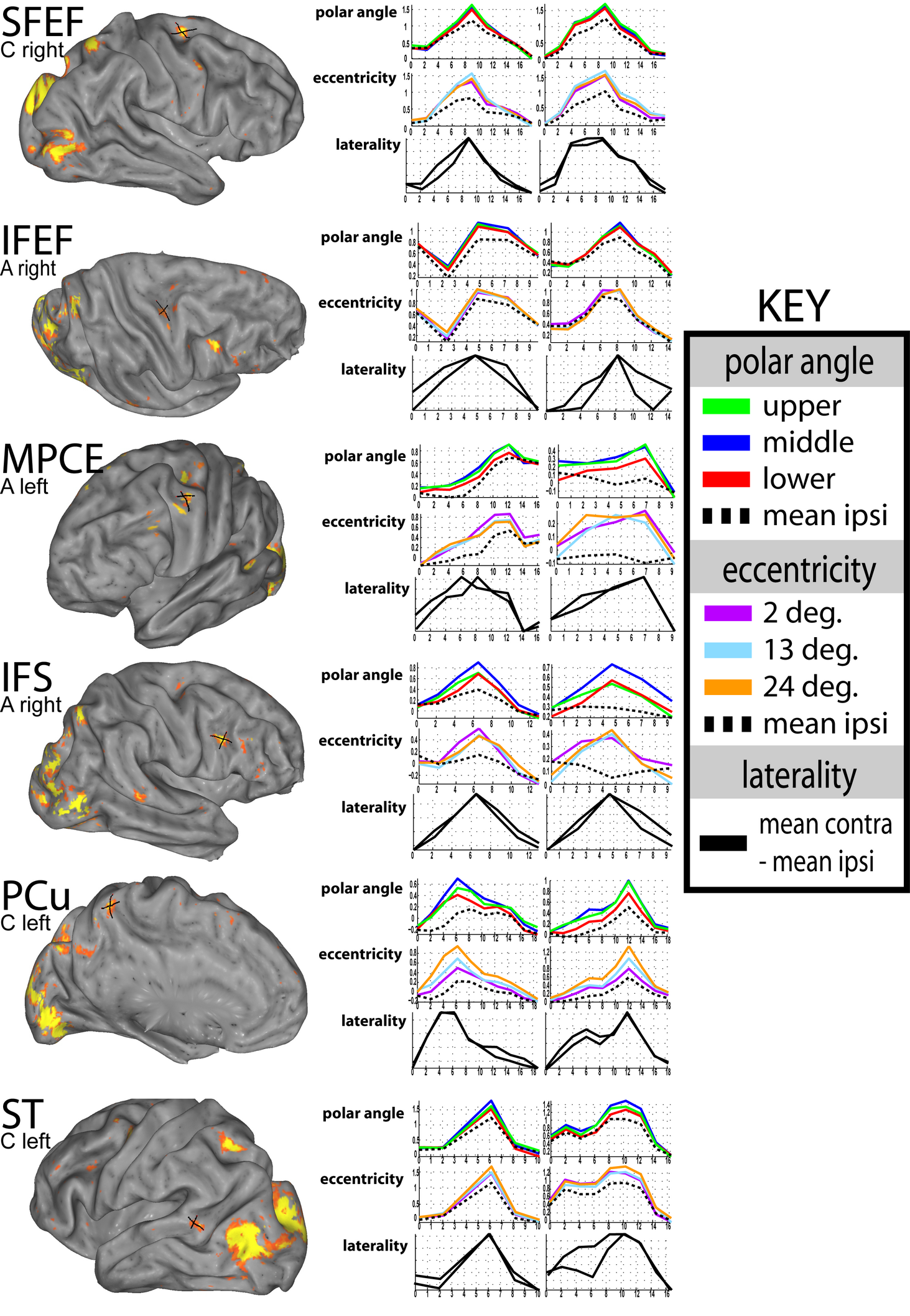

Supplement: Figure S4 — Visual field organization of other extra-occipital regions. We took the most robust example of each region from the two subjects (A and C) who participated in both polar-angle and eccentricity versions of the delayed saccade. We drew two trajectories through each region, and plotted BOLD activity corresponding the three contra-lateral locations in the polar angle (top graph) and eccentricity (middle graph), with mean BOLD response to ipsi-lateral shown by a dotted black line. The bottom of the three graphs for each area shows the mean difference between contralateral and ipsilateral positions for the two data sets (polar angle and eccentricity), with the scale normalized for comparison. (9.28 MB TIF) [file pone.0000452.s004.tif]

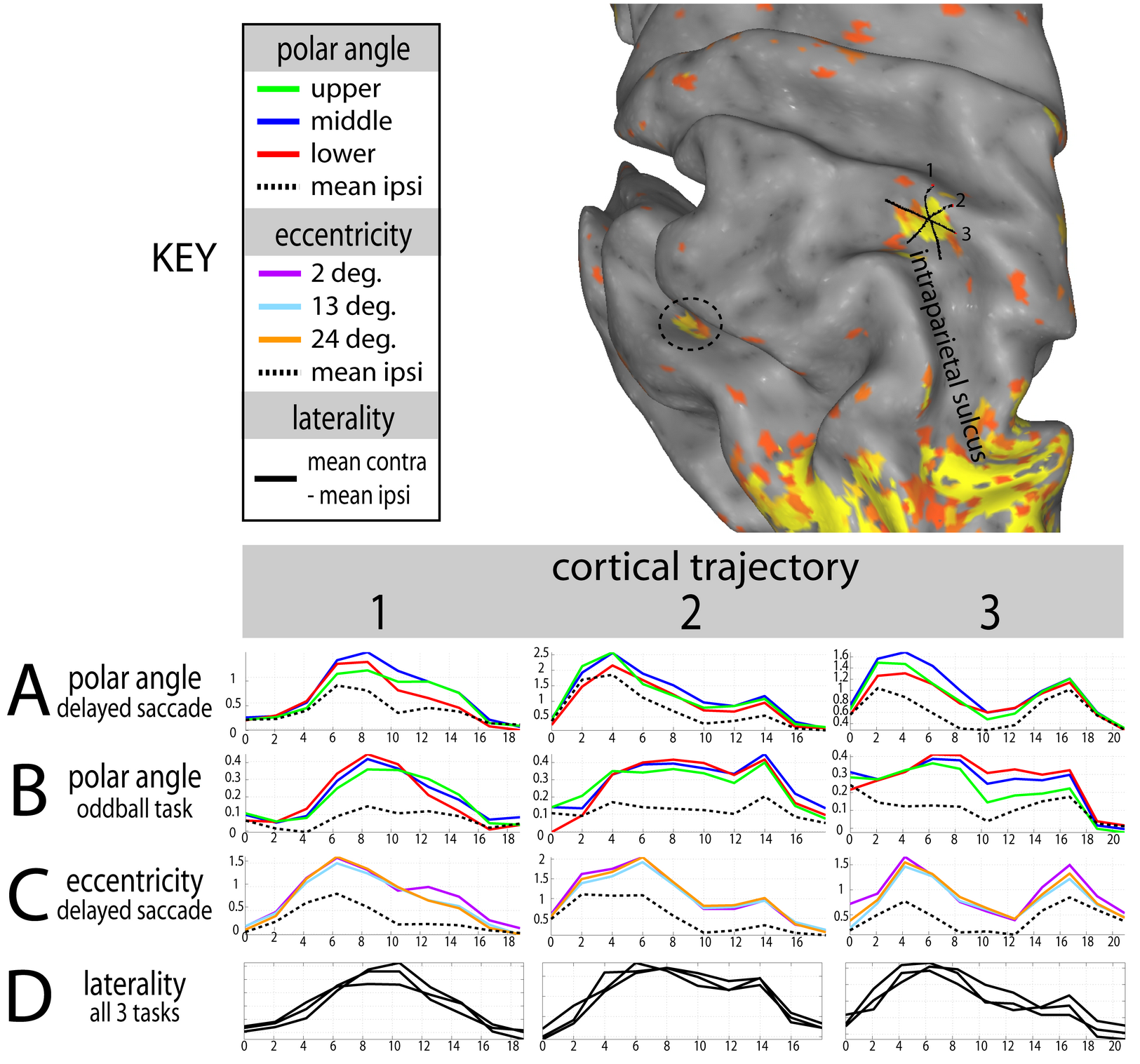

Supplement: Figure S5 — Profiles of activity for cortical trajectories cross-secting area MIPS at three different orientations. An inflated representation of the left hemisphere of subject A is shown, overlayed with a statistical map showing voxels that prefer the contralateral visual field. Trajectories were drawn through area MIPS at three different orientations. The graphs below show the profile of activity along the trajectories, labeled 1–3. (A) shows data from the polar angle version of the delayed saccade task. The three contra-lateral locations are color coded as shown in the key. The dotted black line shows the mean activity due to ipsi-lateral locations. (B) shows data from the oddball task, displayed in the same format (C) shows data from the eccentricity version of the delayed saccade task, with contralateral locations color coded as shown in the key. (D) shows the mean difference between contralateral and ipsilateral field locations for the three tasks, with the scale normalized for comparison. Note that there is evidence of polar angle topography along trajectory (1), with the lower field represented more anterior and the upper field more posterior. This topographic organization was consistent across the two tasks. Nonetheless, BOLD modulation associated with topography was slight compared with the contralateral preference seen for this area. Note the highly consistent profile of contralateral preference for the three tasks illustrated in (D). The dotted circle in the top figure shows the location of area ST in the left hemisphere. (7.40 MB TIF) [file pone.0000452.s005.tif]

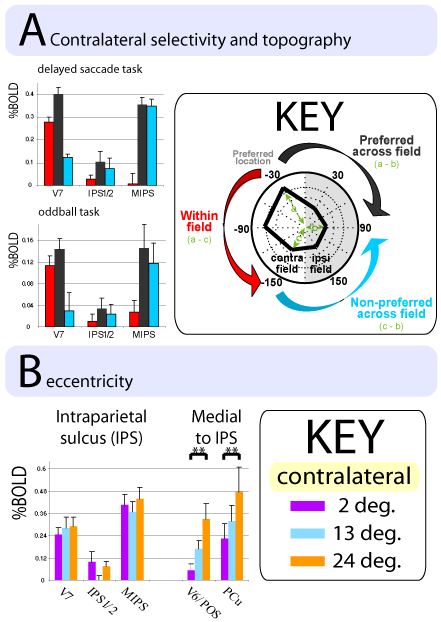

Supplement: Figure S6 — Visuotopic organization of intraparietal sulcus and surrounding cortex. A shows the abrupt change in the degree of topographic organization that occurs between V7 to MIPS. See Figure 6 for further explanation of the graphs. Previous studies have claimed a continuous retinotopic organization stretching along intraparietal sulcus. However the cortical area seperating V7 from MIPS, indicated here as IPS1/2, shows little evidence of contralateral preference or of topographic organization. B illustrates eccentricity organization in intraparietal sulcus and more medial regions. See figure 7 for further explanation of graphs. Regions within intraparietal cortex can be clearly distinguished from more medial regions on the basis of eccentricity preference. (0.86 MB TIF) [file pone.0000452.s006.tif]

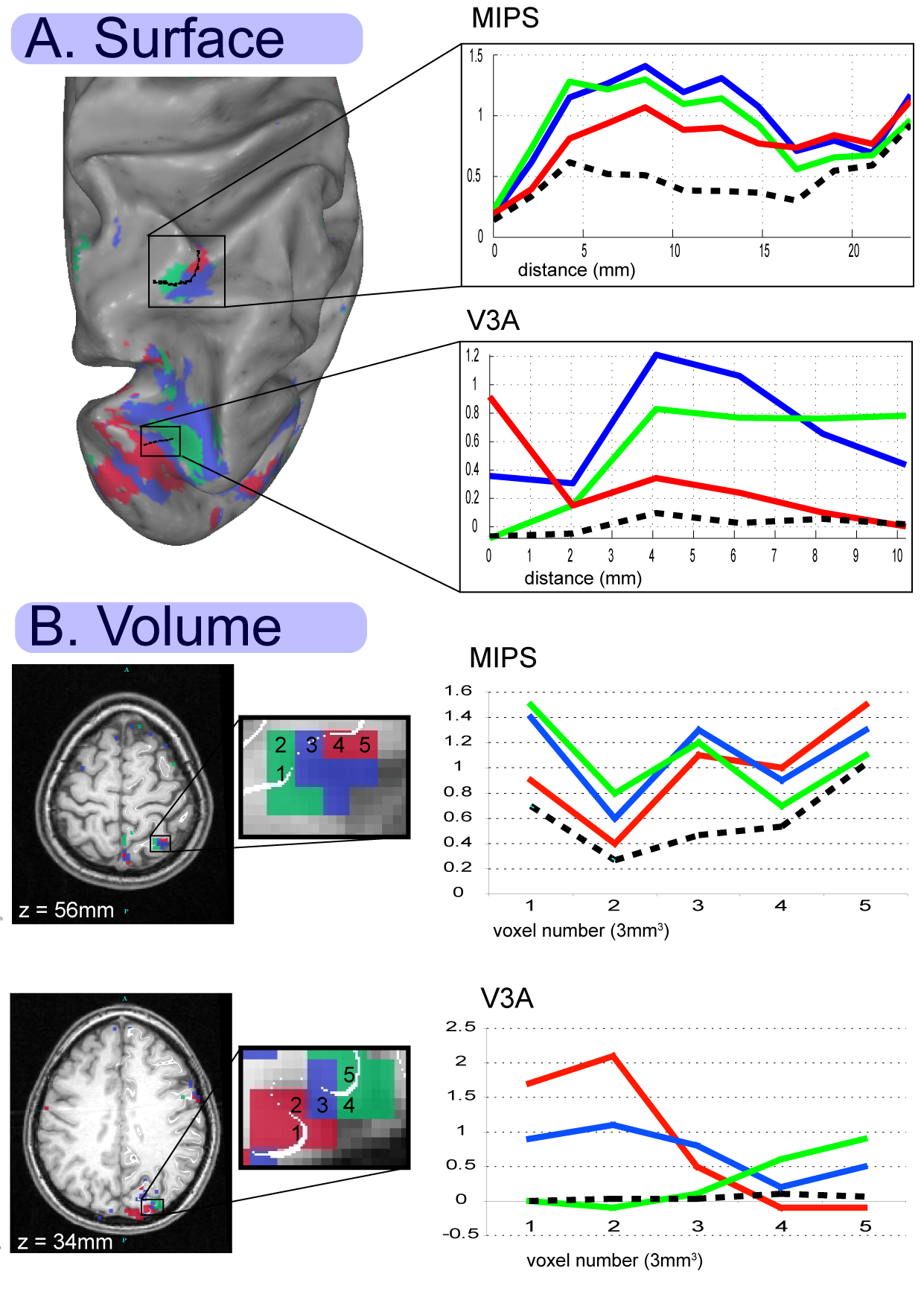

Supplement: Figure S7 — Comparison of best example of MIPS topography (Subject C, right hemisphere) with area V3A in the same hemisphere. The figure illustrates that the reduced topographic organization of area MIPS cannot be accounted for by partial volume effects or by noise-induced spatial smoothing. A shows an inflated representation of the cortical surface with trajectories drawn to optimally capture topographic organization in MIPS and V3A. In area MIPS there is a high degree of correlation between the three contralateral locations. The topography in area V3A is much more clearly defined. The difference between the two areas cannot be attributed to distance, as illustrated by the x-axis of the graphs. In B the graphs trace 5 face-connected voxels that follow the cortical surface (indicated by a white line). Again, response profiles for different visual field positions are highly correlated in MIPS and clearly dissociate in area V3A. The contrast between areas is even more striking in the majority of cases, in which MIPS had no discernable topography. (6.56 MB TIF) [file pone.0000452.s007.tif]
