# Supplementary material for: Changing Human Visual Field Organization from Early Visual to Extra-Occipital Cortex
Source: PLoS One. 2007 May 16;2(5):e452. doi: 10.1371/journal.pone.0000452 (PMC1866221; doi:10.1371/journal.pone.0000452)
Supplement: Supplementary Text S1 — (0.05 MB DOC) [file pone.0000452.s008.doc]

**Cortical line analysis from V1 to IPS**

Illustrations of cortical trajectory analyses are presented for all 8 hemispheres in Figures S1 and S2, analogous to figure 4, panels C-E. Two additional panels are also added. The data in these panels are derived from the data shown in graphs D and E, however they may allow the reader to visualize changes in topographic organization and contralateral preference more clearly. These are explained in the following two sections.

***Magnitude of topography***

To quantify progressive changes in topography over the cortical trajectory we calculated the difference between the preferred contralateral location and the average of the two non-preferred contralateral locations, shown in the panels marked ‘within max’ in figures S1 and S2. A reliable within-field difference score is a necessary condition for topography, but not a sufficient one, since topography additionally requires that different voxels show different within-field preferences in a spatially ordered manner (this additional requirement is clearly satisfied by the responses shown for early visual areas). In early visual areas the within-field difference score varied over the cortical trajectory, rather than remaining at a fixed high level, due to the coarse sampling of the visual field (e.g voxels that responded best to a 60 deg sector, midway between the presented 30 deg and 90 deg sectors, would show a smaller within-field difference score). The maximum within-field difference score decreased in intermediate areas (V3A & V7) and showed the smallest values in parietal cortex (after V7, position 60, Figure 3F). In summary, the maximum within-field difference scores decreased strongly from lower visual areas to parietal cortex, reflecting a progressive decrease in the fineness of topography.

***Magnitude of contralateral preference.***

To quantify contralateral preference along the cortical trajectory, we calculated the difference between the average responses to contralateral and ipsilateral locations, shown in the lower panel marked ‘laterality’ in figures S1 and S2. While contralateral difference scores were low through parts of parietal cortex anterior/dorsal to V7, a clear peak was consistently observed for a small region in the medial intraparietal sulcus which we refer to as MIPS.

***Consistency of results***

In some cases (e.g. subject D, right hemisphere) the thresholded maps showing the preferred contralateral location tiled the cortex from early visual areas to medial intraparietal cortex**,** as reported previously [1,2]. Nonetheless, in each case the profile of BOLD magnitudes revealed a clear shift from early visual areas, with strong within-field difference scores, to parietal cortex, with very small within-field scores. Moreover, in every case we identified an isolated area in medial intraparietal sulcus (MIPS) on the basis of its preference for the contralateral visual field (e.g. Subject D, right hemisphere). Even if the weak topographic signals observed in these areas prove reliable, it is unclear why there should be such profound changes in contralateral preference that fail to correspond to the regional boundaries the topographic organization suggests.

We examined MIPS in our 8 hemispheres and found examples consistent with retinotopic maps, with an ordered representation of the contralateral visual field (e.g. Subject A, left hemisphere, Subject B, right hemisphere and Subject C, right hemisphere). While these findings demonstrate that we can detect likely topographic maps in this part of cortex, our focus here is on the relative strength of topographic vs. non-topographic signals. Even in the above cases, within-field difference scores in MIPS were small compared with the contralateral difference scores or the within-field difference scores in occipital cortex. Therefore, visual field organization in MIPS predominantly reflected contralateral organization rather than topographic organization, even in cases where topography appeared to be present (for illustrations, see figure S5 which shows topography in Subject A, left hemisphere; and figure S7, which shows topography in Subject C, right hemisphere).

The results for MIPS did not depend on the particular axis chosen for the cortical trajectory analysis. Figure S5 shows that very small within-field preferences, coupled with clear contralateral preferences, were observed in MIPS along a range of axes.

***Correlation between contralateral locations along the cortical trajectory*.**

To quantitatively confirm the visual impressions from the cortical trajectory analyses, we calculated the correlation coefficient between BOLD magnitudes due to the middle and lower contralateral positions, the two positions represented in all quarter-field and half-field representations in dorsal occipito-parietal cortex (corresponding to the red and blue lines). The correlation was computed separately for early visual areas, areas V3A and V7, and parietal cortex, and separately for each hemisphere (see Figures S1 and S2). In early visual areas, the mean correlation (averaged across hemispheres) was negative (mean r=-0.25). In areas V3A and V7 there was a small positive correlation (mean r=0.13). In parietal cortex (from the end of V7 to the end of MIPS) there was a very strong positive correlation (mean r=0.95) indicating much less location selectivity. To test for the statistical reliability of this trend, we conducted a random-effects ANOVA across hemispheres with region coded as three levels: early visual, V3A & V7, and parietal. There was a highly significant main effect of region (F[2,14]=26, p<0.001). Post-hoc t-tests indicated that this effect was largely driven by the high correlation seen in parietal cortex as opposed to the other two levels (parietal vs. V3A & V7: t(7)=6.9, p<0.001; parietal vs. early visual: t(7)=7.1, p<0.001; V3A & V7 vs. early visual: t(7)=1.9, p=0.1). Therefore, higher-order areas showed increasingly correlated responses to contralateral polar angles, reducing the degree of topography.

***Correlation between contralateral and ipsilateral locations*.**

We calculated the correlation coefficient between BOLD magnitudes due to the ipsilateral and contralateral positions (see correlation of contralateral sectors above). The correlation was modest and positive in early visual areas (mean r=0.26), but progressively increased in V3A & V7 (mean r=0.58) and parietal cortex (mean r=0.91). This trend was statistically reliable, as indicated by a random-effects ANOVA (F[2,14]=10.3, p=0.002). Again, post-hoc t-tests indicated that this effect was largely driven by the high correlation seen in parietal cortex as opposed to the other two levels (parietal vs. V3A & V7: t(7)=4.1, p=0.005; parietal vs. early visual: t(7)=5.0, p<0.001; V3A & V7 vs. early visual: t(7)=1.6, p=0.15). Therefore, higher-order areas showed increasingly correlated responses to ipsilateral and contralateral locations.

Ruling out effects of partial volume averaging

An important concern is that the limited spatial resolution of fMRI may hinder detection of small topographic maps. Partial volume averaging could lead to a misrepresentation of the degree of BOLD modulation associated with topographic versus contralateral organization, when maps are sufficiently small in size. For instance, if upper and lower contralateral visual field positions are represented so close to each other on the cortical surface that they fall within the same voxel, then this would produce an artificially low estimate of topographic modulation (red bars in Figure 5), and an artificially high estimate of non-topographic contralateral preference (blue bars in Figure 5). However, this explanation can be clearly ruled out for area MIPS. As illustrated in Figures 4, S1 and S2, the spatial extent of MIPS was approximately equivalent to the length of the polar angle axis in V3. Nonetheless area V3 demonstrated clear topography, with clearly dissociated responses to horizontal and lower vertical meridians. Figure S7 compares changes in the response to contralateral stimulus locations in MIPS and V3A as a function of the distance in mm over the cortical surface. Even though we selected the clearest case of topography in MIPS from the eight hemispheres, much larger within-field preferences were observed in V3A over an even shorter cortical distance. The V3A BOLD response to the lower-field location, for example, changed from almost 1% to the level of the ipsilateral response over only a 2 mm cortical distance in the absence of any change to the horizontal meridian location (i.e. note the x-axis from 0 to 2 mm). Similarly the response to the horizontal meridian location changed from roughly 0.35% to 1.2% over only a 2 mm distance in the absence of a changed response to the lower-field location (i.e. note the x-axis from 2 to 4 mm). Therefore, even small changes in cortical distance produced disparate changes to locations in the contralateral field in topographic areas. Similar effects were noted when changes in response were measured to adjacent voxels in transverse slices that cross-sected these regions (Figure S7B). A change of one voxel (i.e. from voxel #2 to #3) produced a substantial change in the response to the lower field location and at best weak changes in the response to the horizontal meridian and upper-field locations. In contrast, responses in MIPS were highly correlated over voxels. These analyses show that the observed differences in topographic organization between V3A and MIPS cannot be accounted for by distance either on the surface representation or in the volume.

Finally, partial volume effects cannot explain any supposed discrepancy between our findings and findings from earlier phase-encoding studies. The 3.25 mm cubic voxels of the current study are comparable to those used in phase-encoding studies (Sereno et al., 2000: 3 x 3 x 4 mm; Silver et al. 2005: 2.5-3.44 mm in-plane x 3 mm out of plane; Schluppeck et al. 2005: 3 x 3 x 3 mm; Hagler & Sereno 2006: 3 x 3 x 3 mm or 3.75 x 3.75 x 3.8 mm).

**Normalized group-averaged measures of topography and contralateral preference across areas**

We computed a normalized measure of contralateral preference, the laterality index, in order to compare preferences across areas. The laterality index for a region was computed as the average contralateral response minus the averaged ipsilateral response, divided by the average contralateral response. The index is shown in the top panel of Figure S3 and followed the expected pattern, with values of about 1.0 in V1-V3, reflecting no ipsilateral response, 0.6 - 0.8 in intermediate visual areas such as V7 or LO/MT, and 0.4 - 0.6 in MIPS and five of the other six extra-occipital areas, reflecting an ipsilateral response that was approximately half as strong as the contralateral response. Therefore, while these extra-occipital areas showed a clear contralateral preference, the ipsilateral response was substantial. Interestingly the precuneus (PCu) had a laterality index of 0.8, comparable to intermediate visual areas, indicating only a small ipsilateral response.

We also computed a normalized measure of the degree of topography, the within-field index, displayed in the bottom panel of Figure S3. The within-field index for a region was computed as the average response to the preferred location in the contralateral field minus the average response to the two non-preferred contralateral locations, divided by the average response to the preferred location. The index followed the expected pattern. High values were observed in V1-V3, but then fell off in intermediate and high-level visual areas. MIPS and the other six extra-occipital areas showed values of only 0.10 or less, reflecting poor topography.

Both the laterality and within-field indices showed strong concordance across tasks. In summary, contralateral preference was far more robust than topographic organization in all extra-occipital regions.

Putative regions IPS1 and IPS2

We defined a region IPS1/2 on the basis of anatomical and functional considerations, as follows. This region comprised that portion of the medial bank of intra-parietal sulcus, from the fundus of the sulcus to the gyrus medial to it, that lay between area V7 and area MIPS. This location corresponds to that described in previous publications [1,2]. Unlike other regions, this region was not thresholded to only include voxels with significant contralateral preference.

Figure S6A illustrates that, even within the intraparietal sulcus, a shift is evident from a clear topographic organization in V7 to a largely non-topographic contralateral organization in MIPS. The region IPS1/2 lay between these two areas. As shown in figure S6A, IPS1/2 demonstrated only a weak preference for the contra-lateral visual field, and no consistent evidence of topography. Our inability to see clear visual field organization in this region was not due to lack of signal, since in the polar angle tasks, the mean BOLD activity in this area (average of all six eccentric locations compared with resting fixation) was at the same level as for V7.

Figure S6B shows that intraparietal sulcus could be distinguished from more medial occipital and parietal cortex on the basis of eccentricity preference. As shown in Figure 6B, MIPS and V7 showed very similar responses to the three eccentricities tested, whereas neighboring regions which lay primarily on the medial surface, V6/POS and PCu, showed a preference for more eccentric locations. None of these regions showed evidence of topographic eccentricity organization, nor were these different areas contiguous in individual surfaces.

**References**

1. Schluppeck D, Glimcher P, Heeger DJ (2005) Topographic organization for delayed saccades in human posterior parietal cortex. J Neurophysiol 94: 1372-1384.

2. Silver MA, Ress D, Heeger DJ (2005) Topographic maps of visual spatial attention in human parietal cortex. J Neurophysiol 94: 1358-1371.
