# Supplementary material for: Changing Human Visual Field Organization from Early Visual to Extra-Occipital Cortex
Source: PLoS One. 2007 May 16;2(5):e452. doi: 10.1371/journal.pone.0000452 (PMC1866221; doi:10.1371/journal.pone.0000452)
Supplement: Supplementary Text S2 — (0.03 MB DOC) [file pone.0000452.s009.doc]

**Anatomical Landmarks for Areas:**

The positions of the seven areas sensitive to visual field location found in each subject are described below in relation to sulcal and gyral landmarks on the undistorted cortical surface (See also Figure S5).

**MIPS** The location of area MIPS (medial intraparietal sulcus) showed some variability, depending on the degree of interruption of intraparietal sulcus and size of superior parietal sulcus [1]. It was found either directly on the medial bank of intraparietal sulcus (more frequent on the left) or on the anterior bank of a small sulcus which extended medially from the intraparietal sulcus (the second variation being similar to the contralateral map described by Sereno, Pitzalis & Martinez (2001)). In some subjects this sulcus was located immediately posterior to the post-central sulcus, such that MIPS extended onto the gyrus that became the posterior bank of the post-central sulcus. In other subjects the superior parietal sulcus was more pronounced and extended dorsally. In these cases, MIPS extended onto the gyrus that formed the posterior bank of superior parietal sulcus, and lay some way posterior to the post-central sulcus. The anatomical location was broadly similar in the two hemispheres.

**PCu** Area PCu (precuneus) was found on the superior portion of the medial surface of the superior parietal lobule (precuneus), clearly anterior to the parietal occipital sulcus. It was generally stronger on the more anterior gyrus of the precuneus, immediately posterior to the superior tip of the marginal branch of the cingulate sulcus and anterior to the superior parietal sulcus. It could be clearly distinguished from MIPS and from a medial higher visual region lying in the fundus of the parietal occipital sulcus (described here as V6/POS, see methods). The anatomical location was similar for the two hemispheres.

**ST** Area ST (superior temporal) was located at the posterior end of the superior temporal sulcus, inferior to the posterior tip of the sylvian fissure. In the left hemisphere it was always located in the fundus of the main sulcus. In the right hemisphere it was found in the superior segment of the superior temporal sulcus and/or (where the superior segment did not connect directly to the main sulcus) on the superior temporal gyrus. Thus the right hemisphere location was somewhat more superior and lateral to the left hemisphere location.

**IFEF** At the junction of the Pre-central and Superior frontal sulci, a larger area was found that typically lay just inferior to the superior frontal sulcus within the pre-central sulcus. Since the role of this anatomical location in eye movements is well established, we refer to it as area IFEF (inferior frontal eye fields). The anatomical location was similar for the two hemispheres, although there was a tendency for it to lie more on the posterior bank of the sulcus in the left hemisphere.

**SFEF** An apparently distinct but smaller area was also evident at the junction of Pre-central and Superior frontal sulci, just superior to IFEF, and lying either within the Superior frontal sulcus or on its superior bank. We refer to this area as SFEF (superior frontal eye fields). The anatomical location was similar for the two hemispheres.

**MPCe** Area MPCe (middle pre-central sulcus) was located on the posterior bank of the pre-central sulcus, very close to the interruption between the superior and inferior limbs of the precentral sulcus, and just dorsal to its junction with the inferior frontal sulcus.

**IFS** Area IFS (inferior frontal sulcus) was located at or near the deepest fundus of the inferior frontal sulcus, approx 2 cm anterior from the junction with the pre-central gyrus. The anatomical location was similar for the two hemispheres.

1. Ono M, Kubik S, Abernathey CD (1990) Atlas of the cerebral sulci. Stuttgart; New York: G. Thieme Verlag; Thieme Medical Publishers. xiv, 218 p. p.
