# Supplementary material for: Changing Human Visual Field Organization from Early Visual to Extra-Occipital Cortex
Source: PLoS One. 2007 May 16;2(5):e452. doi: 10.1371/journal.pone.0000452 (PMC1866221; doi:10.1371/journal.pone.0000452)
Supplement: Supplementary file 4 [file pone.0000452.s003.doc]

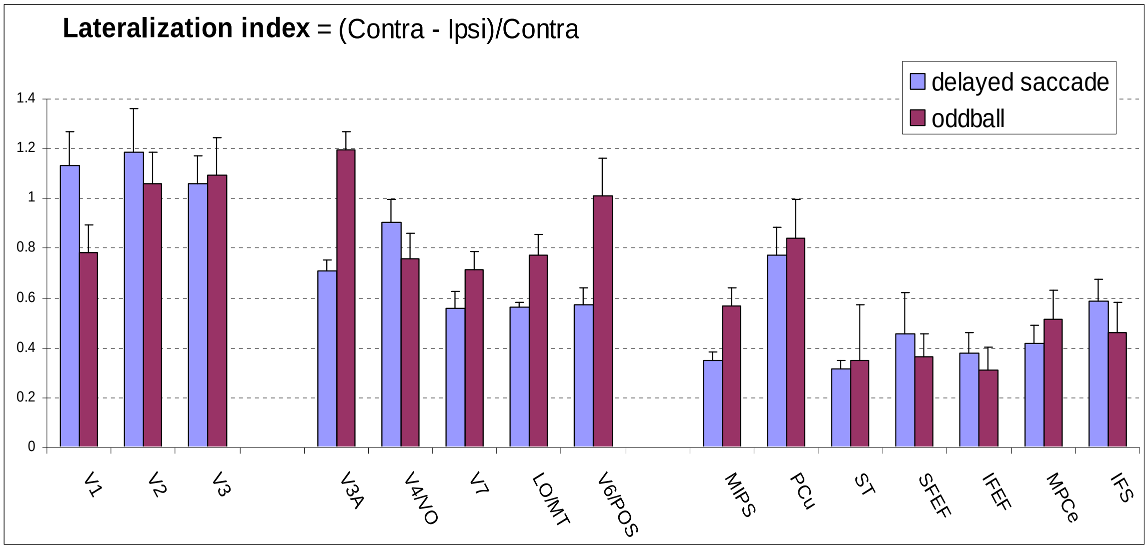

Lateralization index = (Contra - Ipsi)/Contra
delayed saccade
oddball
1.4
1.2
1
0.8
0.6
0.4
0.2
0
V1
V2
V3
V3A
V4/VO
V7
LO/MT
V6/POS
MIPS
PCu
ST
SFEF
IFEF
MPCe
IFS

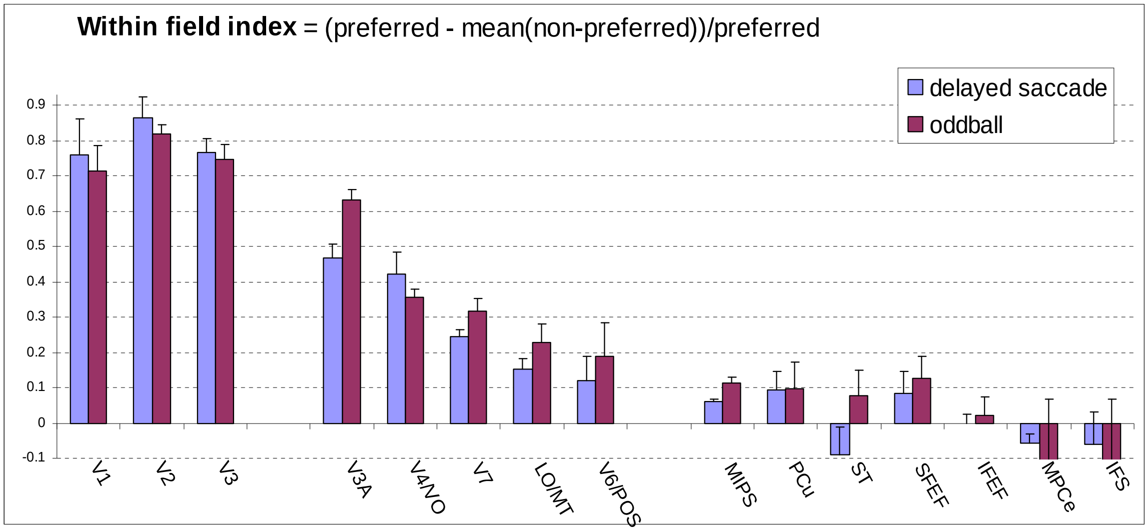

Within field index = (preferred - mean(non-preferred))/preferred
delayed saccade
oddball
0.9
0.8
0.7
0.6
0.5
0.4
0.3
0.2
0.1
0
-0.1
V1
V2
V3
V3A
V4/VO
V7
LO/MT
V6/POS
MIPS
PCu
ST
SFEF
IFEF
MPCe
IFS
